# Identification and Genomic Characterization of a Novel Teseptimavirus Species (vB_EcoP_S37A) and a Mosigvirus Phage (vB_EcoM_S37) Infecting Swine Extraintestinal Pathogenic *Escherichia coli*

**DOI:** 10.3390/vetsci13090943

**Published:** 2026-09-10

**Authors:** Lu Ran, Shenghui Wan, Kexun Lian, Yan Liang, Pei Zheng, Yonggang Qu

**Affiliations:** 1 College of Animal Science and Technology, Shihezi University, Shihezi 832003, China; 20242113054@stu.shzu.edu.cn (L.R.);; 2 Xinjiang Tecon Animal Husbandry Technology Co., Ltd., Changji 831399, China

**Keywords:** extraintestinal pathogenic *Escherichia coli*, bacteriophage, biological characterization, whole-genome analysis, novel *Teseptimavirus* species

## Abstract

The emergence of antimicrobial resistance in extraintestinal pathogenic *Escherichia coli* (ExPEC) necessitates alternative therapies for swine. Two lytic phages, vB_EcoM_S37 and vB_EcoP_S37A, were isolated from swine wastewater and characterized for morphology, stability, host range, antibacterial efficacy, and whole-genome features. Optimal multiplicities of infection were 0.01 and 0.1, respectively. Both phages remained stable at pH 4.0–11.0 and 40–60 °C. vB_EcoM_S37 lysed 43.55% (27/62) of tested *E. coli* strains, with a 5-min latent period and 1173 PFU/cell burst size, and suppressed bacterial growth for 12 h in vitro. vB_EcoP_S37A lysed 6.45% (4/62) of strains, exhibited a 10-min latent period and 210 PFU/cell burst size, and maintained inhibition for 8 h. Genomic analysis revealed that both phages have linear dsDNA genomes devoid of virulence or antibiotic resistance genes. vB_EcoM_S37 (173,927 bp, 37.42% GC) belongs to the genus *Mosigvirus* (family *Straboviridae*), whereas vB_EcoP_S37A (38,725 bp, 48.64% GC) represents a novel species within the genus *Teseptimavirus* (family *Autotranscriptaviridae*). These findings support both phages as stable, safe, and promising candidates for non-antibiotic biocontrol of ExPEC infections in livestock.

## Simple Summary

Antibiotic-resistant bacteria are a growing threat in pig farming, making infections harder to treat. This study discovered two viruses—called bacteriophages—that specifically kill harmful *Escherichia coli* bacteria from pig farm wastewater. Importantly, one of these viruses represents a completely new species never described before, expanding our understanding of viral diversity in nature. The same virus also produces a clear halo around its plaques, suggesting it may use an unusual mechanism to break through bacterial cell walls—a feature that offers new insights into how viruses interact with their hosts. The two viruses, despite attacking the same bacterium, show very different strategies: one has a larger genome and can infect many *E. coli* strains, while the other has a smaller, more streamlined genome and a much narrower host range. This contrast provides a unique opportunity to study how viruses evolve to balance speed, efficiency, and host adaptability. Both viruses are safe, stable, and do not carry resistance genes or toxins. Their complementary properties make them promising candidates for developing alternative treatments against drug-resistant infections in pigs, while also serving as valuable tools for basic phage research.

## 1. Introduction

*Escherichia coli* is a major bacterial pathogen responsible for both intestinal and extraintestinal infections in humans and animals. Based on pathogenicity, *E. coli* can be classified into commensal strains and pathogenic variants, with the latter further subdivided into intestinal pathogenic *E. coli* (IPEC) and extraintestinal pathogenic *E. coli* (ExPEC) [1]. The typical extraintestinal diseases caused by ExPEC include neonatal meningitis, septicaemia, and urinary tract infections [2]. Depending on the site of isolation and host origin, ExPEC can be further categorized into neonatal meningitis-associated *E. coli* (NMEC), septicaemia-associated *E. coli* (SEPEC), urinary tract infection-associated *E. coli* (UPEC), and avian pathogenic *E. coli* (APEC) [3].

In recent years, with the rapid intensification of the swine industry in China, ExPEC has become one of the major pathogens responsible for substantial economic losses in pig production. The infection rate of swine-derived ExPEC has increased dramatically, making this issue increasingly urgent [3]. More importantly, accumulating evidence indicates that ExPEC is capable of cross-host transmission, spreading among pigs, poultry, and humans [4]. This feature suggests that swine-derived ExPEC carries a potential zoonotic risk, thereby posing not only a threat to livestock production but also a significant concern for public health.

Bacteriophages are viruses that specifically infect and lyse bacteria and are widely distributed in natural environments. Owing to their high host specificity, potent bactericidal activity, and favourable safety profile, phages are widely recognized as one of the most promising alternatives for the control of antibiotic-resistant bacterial pathogens. Studies have demonstrated that treatment with phage ΦD effectively cleared *E. coli* 35218 from infected mice within 48 h [5]. In addition, a cocktail of three phages significantly reduced the contamination load of *E. coli* O157:H7 on beef surfaces [6]. Recent advances have further highlighted the potential of phage therapy against ExPEC. Several studies have reported the successful isolation and characterization of phages targeting ExPEC strains of poultry and porcine origin, demonstrating significant reductions in bacterial loads in vitro and in animal models [7,8]. In veterinary applications, phage preparations have been evaluated for the control of colibacillosis in poultry and swine, with promising results in reducing mortality and improving growth performance [9]. However, regulatory frameworks for phage-based veterinary products remain under development, with challenges including standardization of production, stability assessment, and safety evaluation [10].

Against this background, the present study performed systematic biological characterization and whole-genome analysis of two newly isolated *E. coli* phages, vB_EcoM_S37 and vB_EcoP_S37A, to evaluate their antibacterial efficacy and biosafety. The findings are expected to provide a theoretical basis and candidate strain resources for phage-based control of extraintestinal pathogenic *E. coli* infections in animal husbandry.

## 2. Materials and Methods

### 2.1. Bacterial Strains and Sample Collection

*E. coli* S37, a swine-derived extraintestinal pathogenic *E. coli* (ExPEC) strain, was used as the host for phage isolation, purification, and biological characterization. This strain carries resistance genes TEM, CTX-M, tetA, aac(3)IV, aadA, strA, strB, sul1, sul2, and sul3, and exhibits resistance to gentamicin, trimethoprim-sulfamethoxazole, streptomycin, ampicillin, amoxicillin, erythromycin, clindamycin, cefotaxime, and ceftazidime.

The remaining 39 swine ExPEC strains, along with 22 *E. coli* strains from other sources (10 chicken, 7 rabbit, 5 peacock), 5 swine-derived *Klebsiella pneumoniae*, and 2 swine-derived *Proteus mirabilis*, were used for host range determination. All strains were preserved in 20% glycerol at −80 °C.

Faecal and wastewater samples for phage isolation were collected from large-scale pig farms in Changji and Shihezi, Xinjiang.

### 2.2. Phage Isolation and Purification

Following the method described by Wan et al. with slight modifications [11]. *E. coli* S37 was used as the host for phage isolation. The host was cultured to logarithmic phase (OD_600_ ≈ 0.6). One millilitre of bacterial culture and 10 mL of wastewater were added to 200 mL of 2× LB and incubated at 37 °C with shaking for 12 h. The culture was centrifuged at 16,760× *g* for 15 min at 4 °C, and the supernatant was filtered through a 0.22-μm membrane to obtain the phage stock.

The spot test was used to detect lytic phages: 100 μL of the host culture was spread on an LB plate, 10 μL of the phage stock was spotted, and the plate was incubated at 37 °C for 12 h to check for clear plaques. Zones containing plaques were subjected to plaque purification using the double-layer agar method [12]. Single plaques with distinct morphologies were picked and sequentially purified for three rounds to obtain genetically stable phage isolates.

### 2.3. Morphological Observation of Phages

Phage particles were concentrated by PEG 8000 precipitation [13]. The lysate was centrifuged at 7450× *g* for 15 min at 4 °C to remove cell debris, and the supernatant was filtered through a 0.22-μm membrane. The filtrate was sequentially treated with DNase I and RNase A (1 μg/mL each, 37 °C for 1 h) and NaCl (1 M, ice bath for 2 h), followed by centrifugation at 16,760× *g* for 20 min to discard precipitates. PEG 8000 was added to the supernatant at a final concentration of 10% (*w*/*v*). After 1 h on ice, the mixture was centrifuged at 11,640× *g* for 10 min at 4 °C. The pellet was resuspended in SM buffer, extracted with an equal volume of chloroform, and centrifuged at 1050× *g* for 15 min at 4 °C. The aqueous phase was collected as the concentrated phage preparation.

Twenty microlitres of the concentrate was dropped onto a carbon-coated copper grid and allowed to adsorb for 5 min at room temperature, after which excess liquid was removed with filter paper. The grid was stained with 20 μL of 2% (*w*/*v*) phosphotungstic acid (PTA) for 2 min, blotted dry, and dried under an infrared lamp for 10 min. Phage morphology was observed using a transmission electron microscope (HT7700, Hitachi High-Technologies, Tokyo, Japan) operated at 80 kV.

### 2.4. Host Range Determination

The host range of the purified phages was determined against 69 test strains using the spot test [13]. Each strain was cultured in LB broth at 37 °C with shaking to the logarithmic phase (OD_600_ ≈ 0.6). Aliquots of 100 μL of each bacterial culture were evenly spread onto LB agar plates. After the bacterial lawn had dried, 10 μL of each phage stock was spotted onto the seeded agar, with SM buffer used as a negative control. The plates were incubated inverted at 37 °C for 12 h and then examined for clear zones of lysis at the spotted areas. All strains were tested in duplicate.

To confirm productive infection, plaque-forming assays were performed on three representative susceptible strains (i.e., those that had previously shown clear plaques with the phages), following the method described previously [14]. Briefly, 100 μL of serially diluted (10-fold from 10^−4^ to 10^−6^) phage lysate was mixed with 100 μL of an overnight bacterial culture, incubated at 37 °C for 12 h, and the resulting plaques were enumerated. Phage titres were expressed as plaque-forming units per millilitre (PFU/mL).

### 2.5. Determination of the Optimal Multiplicity of Infection

The optimal multiplicity of infection (MOI) of the phage was determined following a modified protocol adapted from Li et al. [15]. The host strain *E. coli* S37 was cultured to logarithmic phase (OD_600_ ≈ 0.6), and the bacterial concentration was adjusted to approximately 10^8^ CFU/mL. One hundred microlitres of the bacterial suspension was mixed with 100 μL of phage suspension in 800 μL of LB broth (total volume 1 mL) to achieve MOIs of 0.000001, 0.00001, 0.0001, 0.001, 0.01, 0.1, 1, 10, and 100. The mixtures were incubated at 37 °C with shaking (180 r/min) for 6 h. After incubation, the cultures were centrifuged at 16,760× *g* for 5 min at 4 °C, and the supernatants were filtered through 0.22-μm membranes. The filtrates were serially diluted 10-fold, and 100 μL of an appropriate dilution was used to determine phage titres by the double-layer agar plate method for each MOI condition. The MOI corresponding to the highest phage titre was defined as the optimal MOI. Each MOI condition was tested in triplicate, and the results were expressed as mean ± standard deviation (SD).

### 2.6. Adsorption Assay and One-Step Growth Curve

To determine the phage adsorption rate, a previously described method was followed with slight modifications [16]. The host strain *E. coli* S37 was mixed with the phage at the optimal multiplicity of infection (MOI) and incubated at 37 °C with gentle shaking. At time points 0, 2, 4, 8, 10, 15, and 20 min, 100-μL aliquots were withdrawn, immediately added to 900 μL of LB broth, and centrifuged. The supernatant was then used to determine the titre of unadsorbed phages by the double-layer agar plate method.

One-step growth curves were determined as previously described [17]. The host strain *E. coli* S37 was cultured to logarithmic phase (OD_600_ ≈ 0.6). The phages were mixed with the host at their respective optimal MOIs and incubated at 37 °C for the optimal adsorption period to allow phage adsorption. The mixture was then centrifuged at 16,760× *g* for 2 min, and the supernatant was discarded. The pellet was washed twice with sterile PBS, resuspended in LB broth, and transferred into 100 mL of pre-warmed LB broth and incubated at 37 °C with shaking at 180 r/min for 60 min. At designated time intervals (0, 5, 10, 15, 20, 25, 30, 40, 50, and 60 min), 400-μL aliquots were collected and immediately centrifuged at 16,760× *g* for 5 min at 4 °C. One hundred microlitres of each supernatant was serially diluted 10-fold, and phage titres were determined using the double-layer agar plate method.

The latent period was defined as the time interval between phage adsorption and the first observable increase in phage titre. The burst size was calculated as the ratio of the phage titre at the plateau phase to the initial infected bacterial count [18]. All assays were performed in triplicate, and the results were expressed as mean ± SD.

### 2.7. Thermal and pH Stability Assays

The stability of the phages at different temperatures and pH values was determined as described above [19].

Thermal stability: Equal volumes of phage suspension were dispensed into sterile tubes (1 mL per tube) and incubated in water baths at 40, 50, 60, 70, and 80 °C for 60 min. Samples of 100 μL were taken at 0, 20, 40, and 60 min, immediately serially diluted 10-fold, and phage titres were determined by the double-layer agar plate method. Phages without heat treatment served as controls. Each temperature condition was tested in triplicate.

pH stability: The pH values of LB broth were adjusted to 2.0, 3.0, 4.0, 5.0, 6.0, 7.0, 8.0, 9.0, 10.0, 11.0, 12.0, and 13.0 using 1 M HCl or 1 M NaOH, and sterilized by filtration through 0.22-μm membranes. One hundred microlitres of phage suspension was mixed with 900 μL of LB broth at each pH and incubated at 4 °C for 2 h. After incubation, 100-μL samples were taken, serially diluted 10-fold, and phage titres were determined by the double-layer agar plate method. Phages treated at pH 7.0 served as controls. Each pH condition was tested in triplicate.

All assays were performed in triplicate, and the results were expressed as mean ± SD.

### 2.8. In Vitro Antibacterial Activity Assay

The antibacterial efficacy of the phage was evaluated following a method adapted from Han et al. [20]. The host strain was cultured to logarithmic phase (OD_600_ ≈ 0.6), and the bacterial concentration was adjusted to approximately 10^6^ CFU/mL. The bacterial suspension was mixed with phage suspensions at different MOIs (0.001, 0.01, 0.1, 1, and 10). Each mixture was inoculated at a 1:100 ratio into 25 mL of LB broth. A positive control (host bacteria without phages) and a negative control (LB broth only) were included. All cultures were incubated at 37 °C with shaking (180 r/min) for 24 h. At designated time points (0, 2, 4, 6, 8, 10, 12, 16, 20, and 24 h), 200-μL samples were transferred to a 96-well microplate. The OD_600_ values were measured using a microplate reader. To determine viable bacterial counts, aliquots from the same cultures were serially diluted 10-fold, and 100 μL of each dilution was spread onto LB agar plates. Colonies were counted after incubation at 37 °C for 12–16 h, and the results were expressed as CFU/mL. All assays were performed in triplicate, and the results were expressed as mean ± SD.

### 2.9. Determination of the Inhibitory and Disruptive Effects of Phages on Biofilms

The inhibitory and disruptive effects of the phages on biofilms were determined following a previously described method with slight modifications [21]. The host strain was inoculated into LB broth and incubated overnight at 37 °C with shaking.

Biofilm inhibition assay: The host strain (1.73 × 10^8^ CFU/mL) was diluted 1:100, and 100 μL of the bacterial suspension and 100 μL of phage suspension at different MOIs (0.01, 0.1, and 1) were added to each well of a 96-well cell culture plate. The plates were incubated statically at 37 °C for 12, 24, and 48 h.

Biofilm disruption assay: The host strain (1.73 × 10^8^ CFU/mL) was diluted 1:100, and 200 μL of the bacterial suspension was added to each well of a 96-well plate. The plates were incubated at 37 °C for 48 h to allow mature biofilm formation. After incubation, the supernatant was carefully removed, and 200 μL of phage suspension at different MOIs (0.01, 0.1, and 1) was added to each well. The plates were further incubated at 37 °C for 6, 12, and 24 h.

Biofilm quantification: After incubation, the culture medium was discarded, and each well was washed three times with 200 μL of sterile PBS to remove non-adherent cells. Subsequently, 200 μL of methanol was added to each well for fixation for 15 min. The methanol was then removed, and the plates were air-dried at room temperature. Each well was stained with 200 μL of 0.1% (*w*/*v*) crystal violet for 15 min. The crystal violet solution was aspirated, and the wells were rinsed three times with running water and air-dried. Then, 200 μL of 33% (*v*/*v*) glacial acetic acid was added to each well and incubated for 20 min to solubilize the stained biofilm. The absorbance was measured at 590 nm (OD_590_) using a full-wavelength microplate reader. All experiments were performed in triplicate, and the results were expressed as mean ± SD.

### 2.10. Phage Genome Extraction, Sequencing, and Bioinformatic Analysis

Phage genomic DNA was extracted following the method described by Yang et al. [22]. Briefly, 1 mL of concentrated phage suspension was treated with DNase I and RNase A at a final concentration of 1 μg/mL each for 30 min at room temperature to remove residual host nucleic acids. Proteinase K (final concentration 50 μg/mL) and sodium dodecyl sulfate (SDS, final concentration 0.5%, *w*/*v*) were then added, and the mixture was incubated at 56 °C for 1 h. An equal volume of phenol–chloroform–isoamyl alcohol (25:24:1, *v*/*v*) was added, vortexed, and centrifuged at 16,760× *g* for 5 min. The aqueous phase was collected, and DNA was precipitated with 2 volumes of absolute ethanol at 4 °C for 30 min, followed by centrifugation at 16,760× *g* for 10 min. The pellet was washed, air-dried, and resuspended in 80–100 μL of sterile water. The extracted phage DNA was sent to General Biosystems (Chuzhou, Anhui, China) for whole-genome sequencing.

Whole-genome sequencing was performed on an Illumina NovaSeq 6000 platform, generating 2 × 150 bp paired-end reads. Raw reads were quality-filtered using Fastp v0.23.4. For vB_EcoM_S37, a total of 33,843,060 high-quality reads were obtained (Q20 ≥ 99.81%, Q30 ≥ 98.5%), totaling approximately 5.09 Gb, with an average sequencing coverage of approximately 29,282×. For vB_EcoP_S37A, a total of 48,332,112 high-quality reads were obtained (Q20 ≥ 99.34%, Q30 ≥ 97.38%), totaling approximately 7.28 Gb, with an average sequencing coverage of approximately 187,964×. Genome assembly was performed using SPAdes v3.15.5 with the --careful and --cov-cutoff auto parameters. Genome completeness and the presence of direct terminal repeats were assessed using CheckV v1.0.1. CheckV quality assessment indicated that both genomes were 100% complete with no detected contamination (kmer_freq = 1). For vB_EcoP_S37A, the assembly was processed by circularly permuting the sequence to correctly identify the genome termini.

Genome sequences were compared with other nucleotide sequences using NCBI BLASTn (https://blast.ncbi.nlm.nih.gov/Blast.cgi, accessed on 16 August 2026). Average nucleotide identity (ANIb) was calculated using the JSpeciesWS (https://jspecies.ribohost.com/jspeciesws/, accessed on 24 August 2026) online tool [23]. Genome annotation was performed using Prokka [24], and tRNA genes were predicted using tRNAscan-SE v.2.0 [25,26]. The predicted open reading frames (ORFs) were manually verified by BLASTp searches against the NCBI non-redundant protein database with thresholds of >50% sequence identity and an E-value < 1.0 × 10^−3^ [27]. Multiple genome alignments were generated using Mauve 2.4.0. Antibiotic resistance genes and virulence factors were screened against the CARD database and the VFDB database, respectively. The phage life cycles were predicted using PhageAI (https://app.phage.ai/phages/upload, accessed on 18 August 2026), PhaTYP (https://phage.ee.cityu.edu.hk/phabox, accessed on 18 August 2026), Integron Finder (https://usegalaxy.org/, accessed on 24 August 2026), and CONJscan (https://galaxy.pasteur.fr/, accessed on 24 August 2026). Synteny analysis and visualization of the two phages and their close relatives were performed using Easyfig v2.2.5. PhaRBP was used to predict phage receptor-binding proteins. Conserved domain analysis was performed using the NCBI Conserved Domain Database (CD-Search) to identify functional domains within the phage proteins. Protein secondary structures were predicted using Phyre2 (https://www.sbg.bio.ic.ac.uk/, accessed on 28 August 2026).

The therapeutic potential of the phages was evaluated using PhageLeads (https://globeapp.ku.dk/sws/, accessed on 20 August 2026) [28]. Circular and rectangular proteomic trees were constructed using the ViPTree server [29], including the isolated phages and closely related dsDNA prokaryotic viruses. Phylogenetic trees based on the major capsid protein and the large subunit of terminase were constructed using MEGA 12 with the maximum-likelihood (ML) method, and node support was assessed by 1000 bootstrap replicates.

### 2.11. Statistical Analysis

Phage-related image analyses were performed using ImageJ1.5.4 g. All experiments were conducted in triplicate, and data are presented as mean ± SD. Normality was assessed using the Shapiro–Wilk test. Statistical comparisons were performed using one-way ANOVA followed by Tukey’s HSD post-hoc test in SPSS 26.0. A *p*-value of <0.05 was considered statistically significant. Figures were generated using GraphPad Prism10.1.2.

## 3. Results

### 3.1. Morphological Characterization of Phages

**Figure 1:**
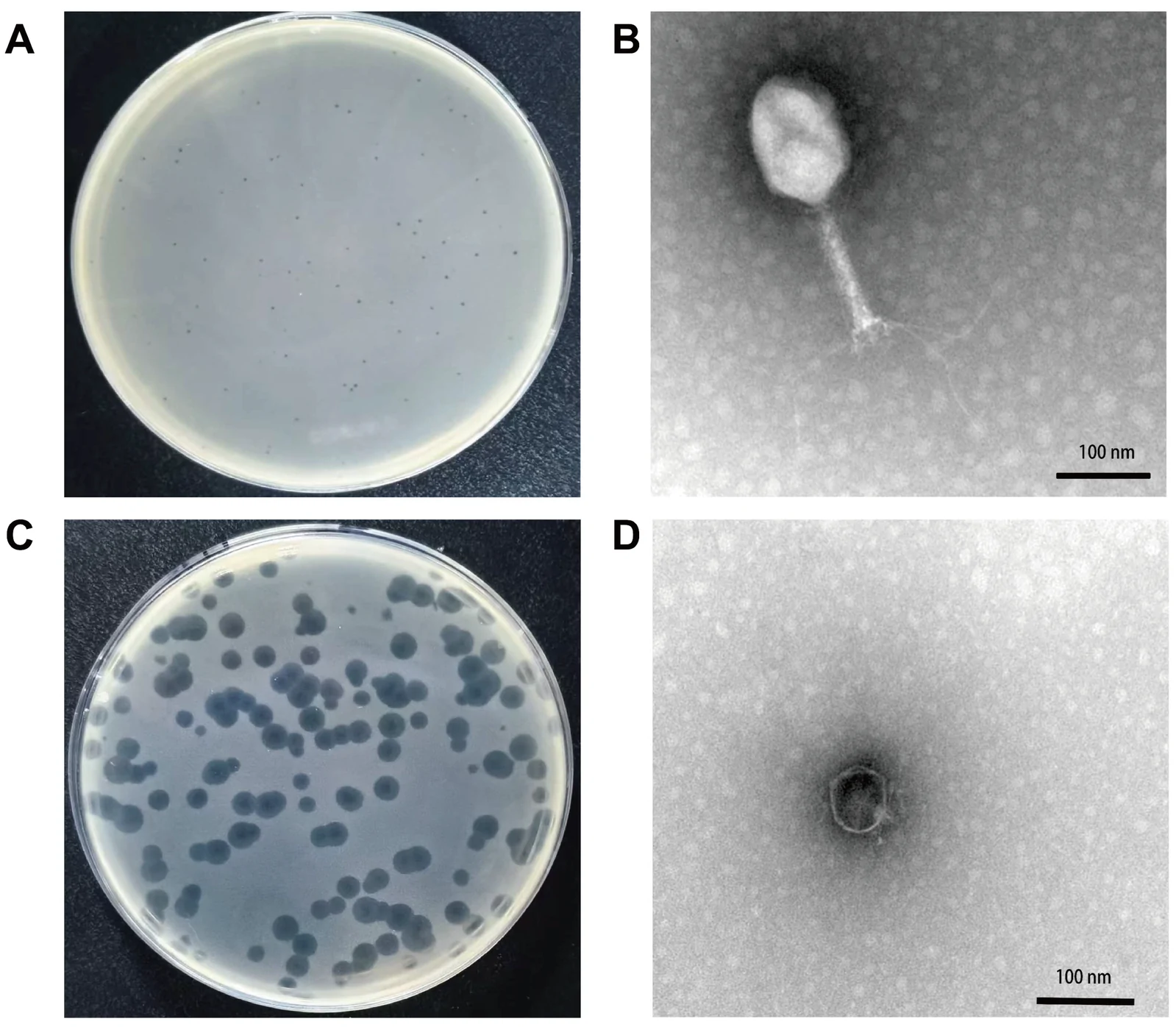
Morphological characteristics of phages vB_EcoM_S37 and vB_EcoP_S37A. (**A**) Plaques of phage vB_EcoM_S37 on LB agar plates; (**B**) transmission electron micrograph of phage vB_EcoM_S37; (**C**) plaques of phage vB_EcoP_S37A on LB agar plates; (**D**) transmission electron micrograph of phage vB_EcoP_S37A.

Using the swine-derived extraintestinal pathogenic *E. coli* S37 as the host, two lytic phages were isolated from wastewater samples. The two phages formed distinct plaque morphologies on LB agar plates: one produced small, clear plaques (Figure 1A), while the other produced plaques with distinct halos (Figure 1C). Transmission electron microscopy further revealed marked differences in tail structure between the two phages: one possessed a contractile tail, whereas the other had a short, non-contractile tail. Based on these morphological characteristics, they were designated vB_EcoM_S37 and vB_EcoP_S37A, respectively.

Transmission electron microscopy revealed that phage vB_EcoM_S37 possesses a contractile tail and an icosahedral head, with a head diameter of approximately 100 ± 2 nm and a tail length of 136.40 ± 2 nm (Figure 1B), consistent with the morphology of the family *Myoviridae* (currently classified within the family *Straboviridae*). Phage vB_EcoP_S37A exhibited a tadpole-like morphology with an icosahedral head (approximately 60 nm in diameter) and a short, non-contractile tail (approximately 17 nm) (Figure 1D), consistent with the typical morphology of the family *Podoviridae* (currently classified within the family *Autotranscriptaviridae*).

### 3.2. Host Range of the Isolated Phages

Phage vB_EcoM_S37 showed lytic activity against 27 of the 62 tested *E. coli* strains (43.55%), including 8 swine, 14 chicken, 3 rabbit, and 2 peacock isolates. Phage vB_EcoP_S37A lysed 4 strains (6.45%), comprising 3 swine and 1 chicken isolate. To confirm productive infection of the spot-test-positive strains, plaque-forming assays were performed on three representative susceptible strains for each phage. All tested strains yielded clear plaques on double-layer agar plates, with titers ranging from 1.5 × 10^7^ to 1.12 × 10^8^ PFU/mL for vB_EcoM_S37 and from 1.07 × 10^5^ to 2.0 × 10^5^ PFU/mL for vB_EcoP_S37A. These results confirm that the spot test results reliably reflected productive lytic infection. Neither phage exhibited lytic activity against *K. pneumoniae* (5 strains) or *P. mirabilis* (2 strains), indicating that their host ranges are restricted to *E. coli* (Table 1).

### 3.3. Optimal Multiplicity of Infection

**Figure 2:**
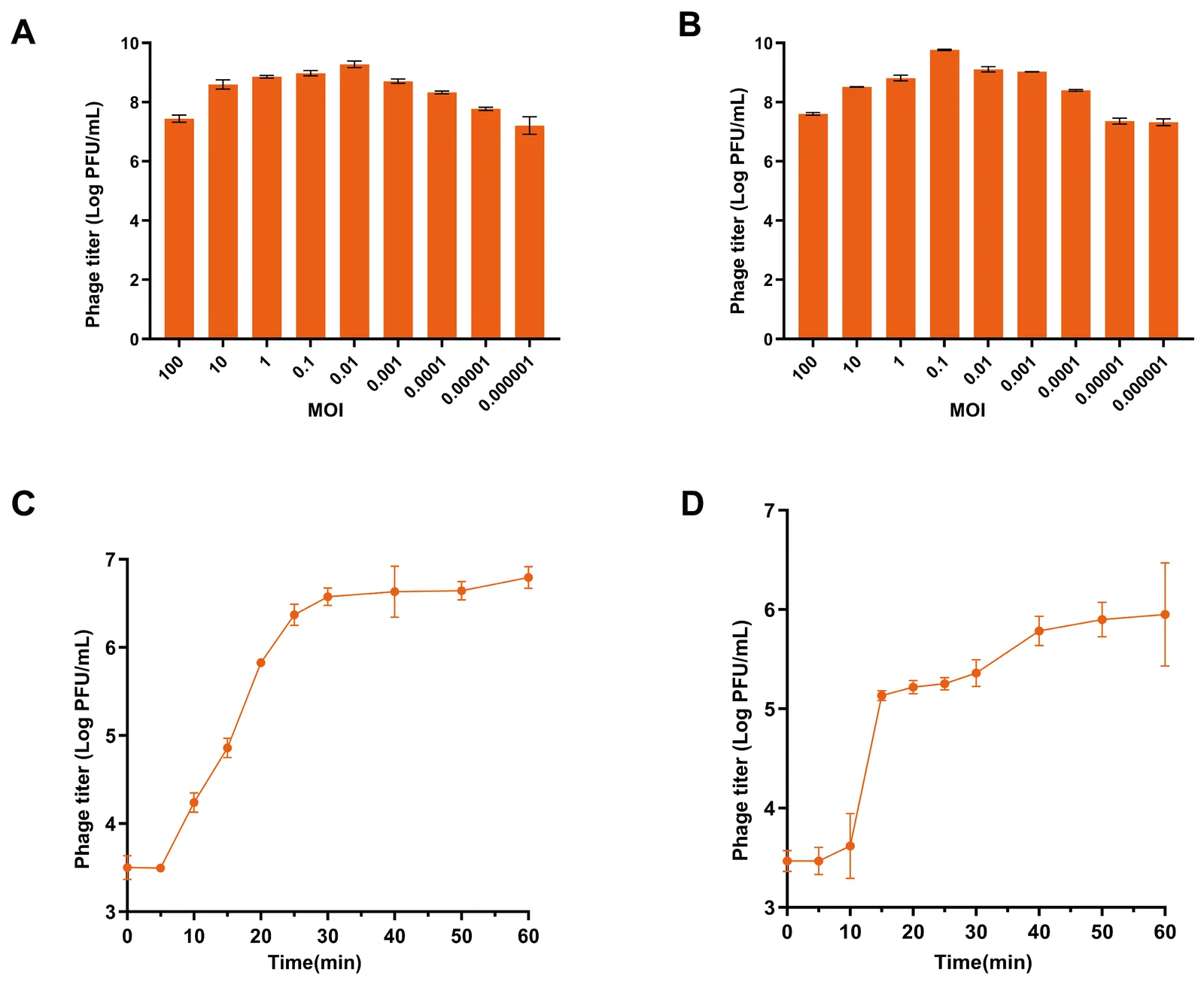
Biological characterization of phages vB_EcoM_S37 and vB_EcoP_S37A. (**A**) Optimal multiplicity of infection (MOI) of phage vB_EcoM_S37. (**B**) Optimal MOI of phage vB_EcoP_S37A. (**C**) One-step growth curve of phage vB_EcoM_S37. (**D**) One-step growth curve of phage vB_EcoP_S37A. Data are presented as mean ± SD from three independent experiments.

The optimal multiplicity of infection (MOI) was determined for both phages. Phage vB_EcoM_S37 exhibited the highest titre of 9.28 log_10_ PFU/mL at an MOI of 0.01 (Figure 2A), while phage vB_EcoP_S37A reached its maximum titre of 9.77 log_10_ PFU/mL at an MOI of 0.1 (Figure 2B).

### 3.4. Adsorption Kinetics and One-Step Growth Curves

The adsorption kinetics of phages vB_EcoM_S37 and vB_EcoP_S37A to *E. coli* S37 were assessed over a 20-min period (Figure S1). vB_EcoM_S37 reached a maximum adsorption rate of 94.78% at 15 min, whereas vB_EcoP_S37A peaked at 77.94% at 8 min, followed by a gradual decrease to 23.53% at 20 min. These results demonstrate that both phages adsorb efficiently to the host, with vB_EcoM_S37 exhibiting significantly higher adsorption efficiency than vB_EcoP_S37A.

One-step growth curve analysis revealed that phage vB_EcoM_S37 had a latent period of 5 min and entered the plateau phase at 30 min post-infection, with an average burst size of 1173 PFU/cell (Figure 2C). Phage vB_EcoP_S37A exhibited a latent period of 10 min, reached the plateau phase at 40 min post-infection, and had a burst size of 210 PFU/cell (Figure 2D).

### 3.5. Thermal Stability

**Figure 3:**
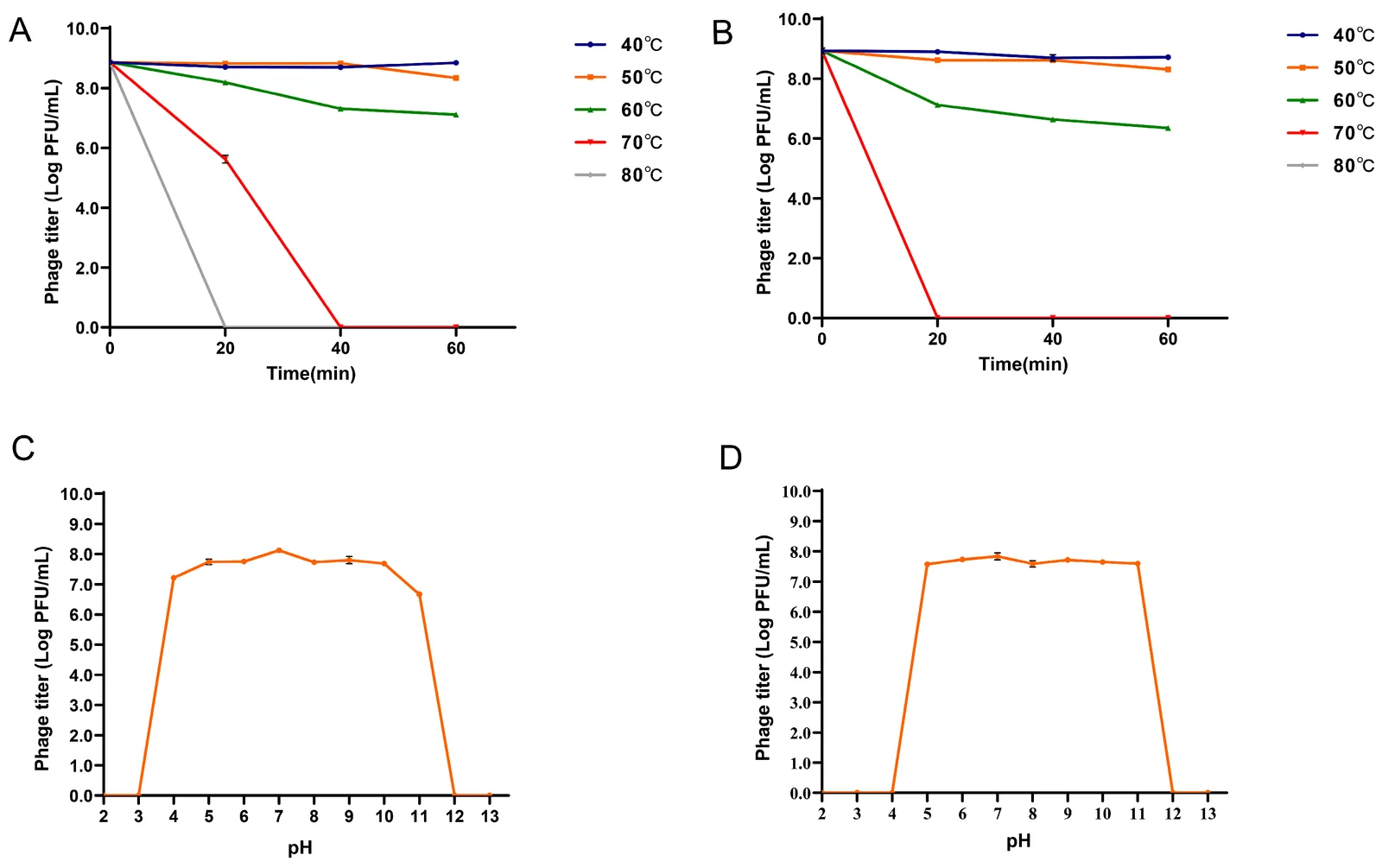
Thermal and pH stability of phages vB_EcoM_S37 and vB_EcoP_S37A. (**A**) Thermal stability of vB_EcoM_S37. (**B**) Thermal stability of vB_EcoP_S37A. (**C**) pH stability of vB_EcoM_S37. (**D**) pH stability of vB_EcoP_S37A. Data are presented as mean ± SD from three independent experiments.

The thermal stability of the two phages was evaluated at different temperatures. Both phages showed no significant change in titre after 60 min of incubation at 40 °C and 50 °C. At 60 °C, the titres of both phages decreased progressively over 60 min, with vB_EcoM_S37 decreasing by approximately 1.8 log_10_ PFU/mL and vB_EcoP_S37A by approximately 2.63 log_10_ PFU/mL. At 70 °C, vB_EcoM_S37 was completely inactivated after 40 min, whereas vB_EcoP_S37A lost viability after 20 min. Both phages were completely inactivated within 20 min at 80 °C (Figure 3A,B). Collectively, these results indicate that both phages possess good thermal stability, with vB_EcoM_S37 exhibiting greater thermostability than vB_EcoP_S37A.

### 3.6. pH Stability

The stability of the two phages was assessed across a range of pH values. Phage vB_EcoM_S37 remained stable at pH 4.0–11.0, but was completely inactivated at pH ≤ 3.0 or pH ≥ 12.0 (Figure 3C). Phage vB_EcoP_S37A exhibited high stability at pH 5.0–11.0, and was completely inactivated at pH ≤ 4.0 or pH ≥ 12.0 (Figure 3D). These results indicate that vB_EcoM_S37 has broader pH stability than vB_EcoP_S37A.

### 3.7. In Vitro Antibacterial Activity

**Figure 4:**
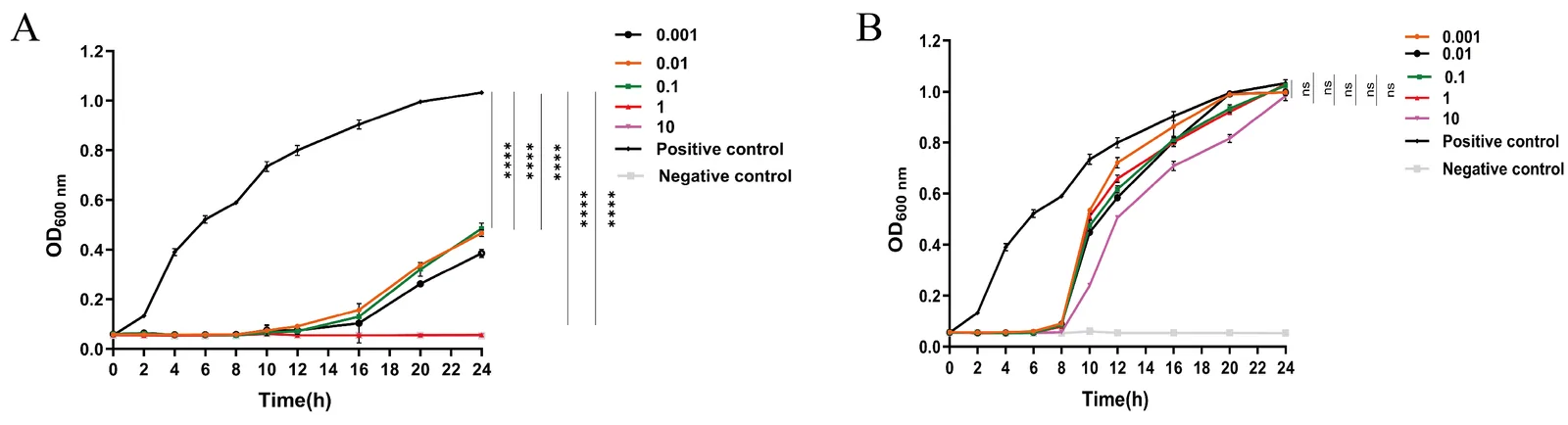
In vitro antibacterial activity of phages vB_EcoM_S37 and vB_EcoP_S37A against the host strain *E. coli* S37. The host strain was mixed with each phage at MOIs of 0.001, 0.01, 0.1, 1, and 10, and incubated at 37 °C with shaking for 24 h; samples were collected at 2-h intervals. (**A**) Antibacterial activity of phage vB_EcoM_S37 (OD_600_). (**B**) Antibacterial activity of phage vB_EcoP_S37A (OD_600_). The positive control was the host culture without phages. Data represent the mean ± SD from three independent replicates. **** *p* < 0.0001; ns, not significant.

Both phages exhibited significant antibacterial activity against the host strain at all MOIs tested (0.001, 0.01, 0.1, 1, and 10). Phage vB_EcoM_S37 almost completely suppressed host bacterial growth within the first 12 h post-infection. Between 8 and 24 h, the OD_600_ values at MOIs 0.001, 0.01, and 0.1 showed a slow increase but remained below the positive control level (Figure 4A); at MOIs 1 and 10, OD_600_ remained nearly unchanged throughout the 24-h period, likely due to sustained suppression at higher MOIs. Phage vB_EcoP_S37A nearly completely inhibited host growth within 8 h, with regrowth observed between 6 and 24 h, although the cell density remained consistently below the control level (Figure 4B). CFU enumeration data are presented in Figure S2. Collectively, phage vB_EcoM_S37 demonstrated superior in vitro antibacterial efficacy compared to vB_EcoP_S37A across the MOI range tested.

### 3.8. Inhibitory and Disruptive Effects of Phages on Biofilms

**Figure 5:**
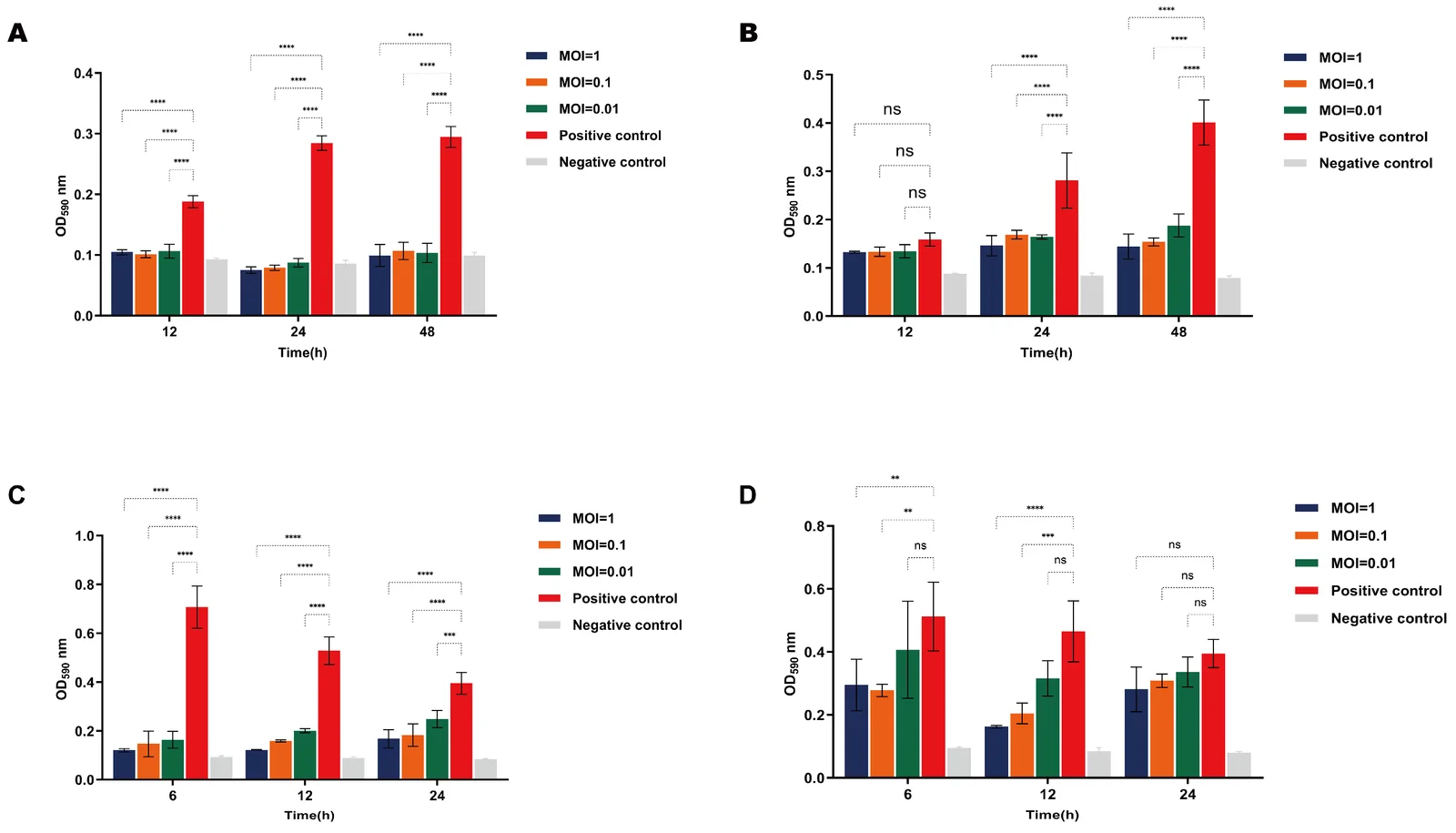
Inhibitory and disruptive effects of phages vB_EcoM_S37 and vB_EcoP_S37A on biofilms. (**A**) Inhibition by vB_EcoM_S37. (**B**) Inhibition by vB_EcoP_S37A. (**C**) Disruption by vB_EcoM_S37. (**D**) Disruption by vB_EcoP_S37A. Biofilm biomass was quantified by crystal violet staining (OD_590_). Data are presented as mean ± SD from three independent experiments. **** *p* < 0.0001,*** *p* < 0.001, ** *p* < 0.01; ns, not significant.

Crystal violet staining assays revealed that both phages inhibited biofilm formation by *E. coli* S37, albeit with distinct efficiencies. vB_EcoM_S37 significantly inhibited biofilm formation across all tested time points (12, 24, and 48 h) and MOIs (0.01, 0.1, and 1), with OD_590_ values markedly lower than the untreated control (*p* < 0.0001) (Figure 5A). In contrast, vB_EcoP_S37A showed no significant effect at 12 h but significantly inhibited biofilm formation at 24 and 48 h (*p* < 0.0001), indicating a time-dependent effect (Figure 5B).

For biofilm disruption, vB_EcoM_S37 significantly disrupted pre-formed biofilms across all conditions (**** *p* < 0.0001) (Figure 5C), whereas vB_EcoP_S37A exhibited no obvious disruption at 12 h, but showed significant activity at 24 h at MOIs of 0.1 and 1 (** *p* < 0.01) (Figure 5D). Collectively, vB_EcoM_S37 demonstrated superior anti-biofilm activity over vB_EcoP_S37A, particularly in eradicating mature biofilms.

### 3.9. Phage Genome Annotation

Phage vB_EcoM_S37 has a genome of 173,927 bp with a GC content of 37.42%, while phage vB_EcoP_S37A has a genome of 38,725 bp with a GC content of 48.64%. Both genomes are linear double-stranded DNA and carry no antibiotic resistance genes or virulence factors (Table S1). The genome sequences have been deposited in the NCBI GenBank database under accession numbers PZ357121 (vB_EcoM_S37) and PZ502562 (vB_EcoP_S37A).

**Figure 6:**
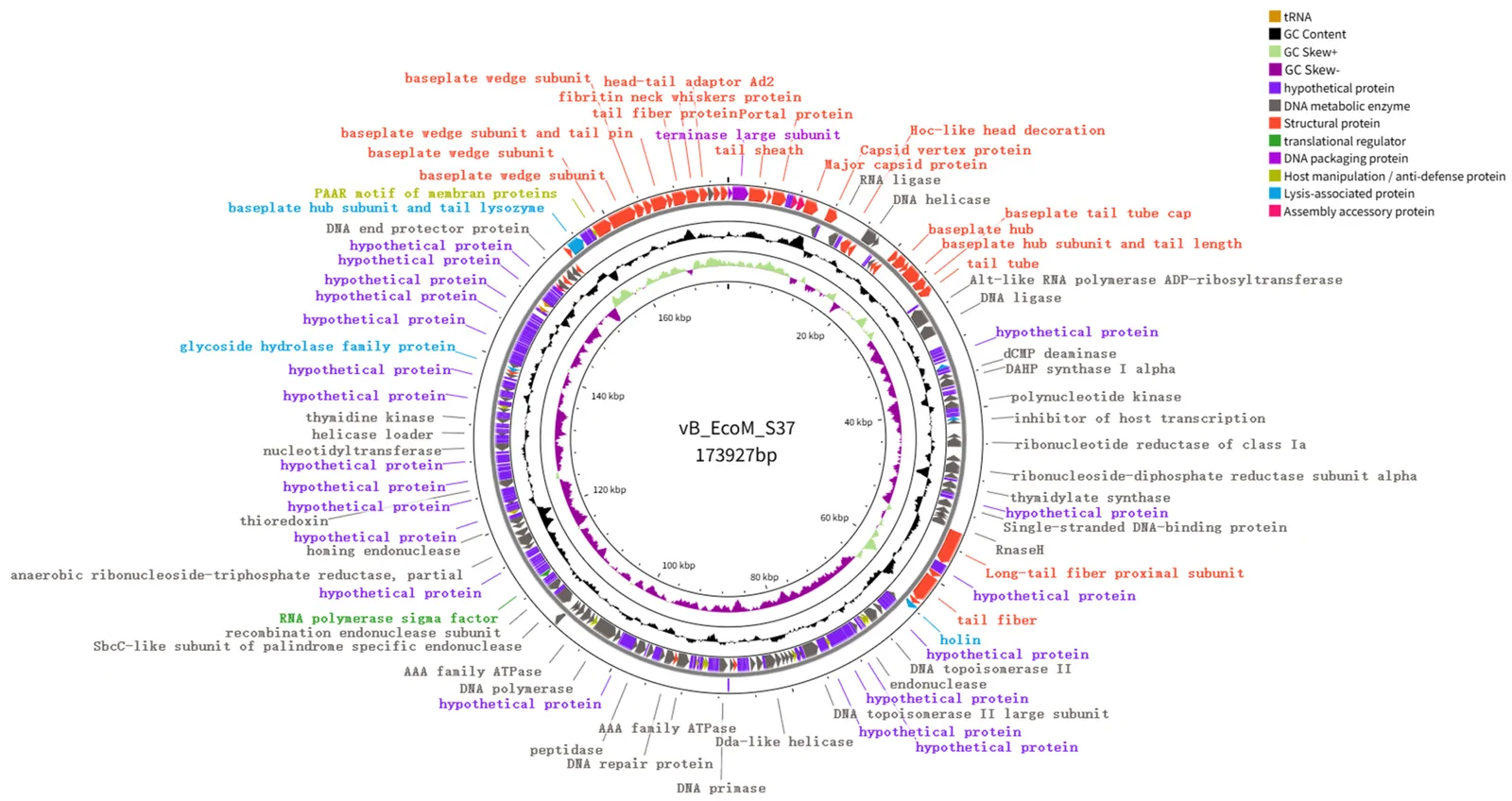
Circular genome maps of vB_EcoM_S37. ORFs are color-coded by functional categories; tRNA genes are marked on the maps.

The genome of vB_EcoM_S37 contains 276 predicted open reading frames (ORFs) distributed on both strands, and two tRNA genes were identified, including tRNA-Met (nt 148,106–148,177, anticodon CAT), and tRNA-Arg (nt 147,227–147,299, anticodon TCT). Among the 276 ORFs, 134 (48.55%) were annotated as known functional proteins and 127 (54.98%) as hypothetical proteins. Functional classification categorized the ORFs into structural proteins (37), translational regulators (1), DNA packaging proteins (2), assembly accessory proteins (3), host manipulation/anti-defense proteins (10), lysis-associated proteins (5), and DNA metabolism and replication proteins (76) (Figure 6). Detailed information on phage protein functions is provided in Table S2. Among the structural proteins, ORF10 and ORF271 encode the major capsid protein and tail fibre protein, respectively. Lysis-associated proteins include ORF45 (rIII lysis inhibitor accessory protein), ORF60 (Rz-like spanin), ORF84 (holin), ORF103 (rIIA lysis inhibitor), ORF229 (glycoside hydrolase family protein), and ORF261 (tail lysozyme). ORF103 encodes the rIIA lysis inhibitor, which protects the bacterial inner membrane during late infection to delay lysis and allow more time for progeny phage assembly. DNA metabolism and replication genes include DNA polymerase (ORF150), primase (ORF127), helicases (ORF19, ORF20), endonucleases (ORF54, ORF63, ORF66, ORF99, ORF186, ORF219), and topoisomerases (ORF93, ORF105). In addition, ORF132 was predicted to encode an immunity to superinfection protein.

**Figure 7:**
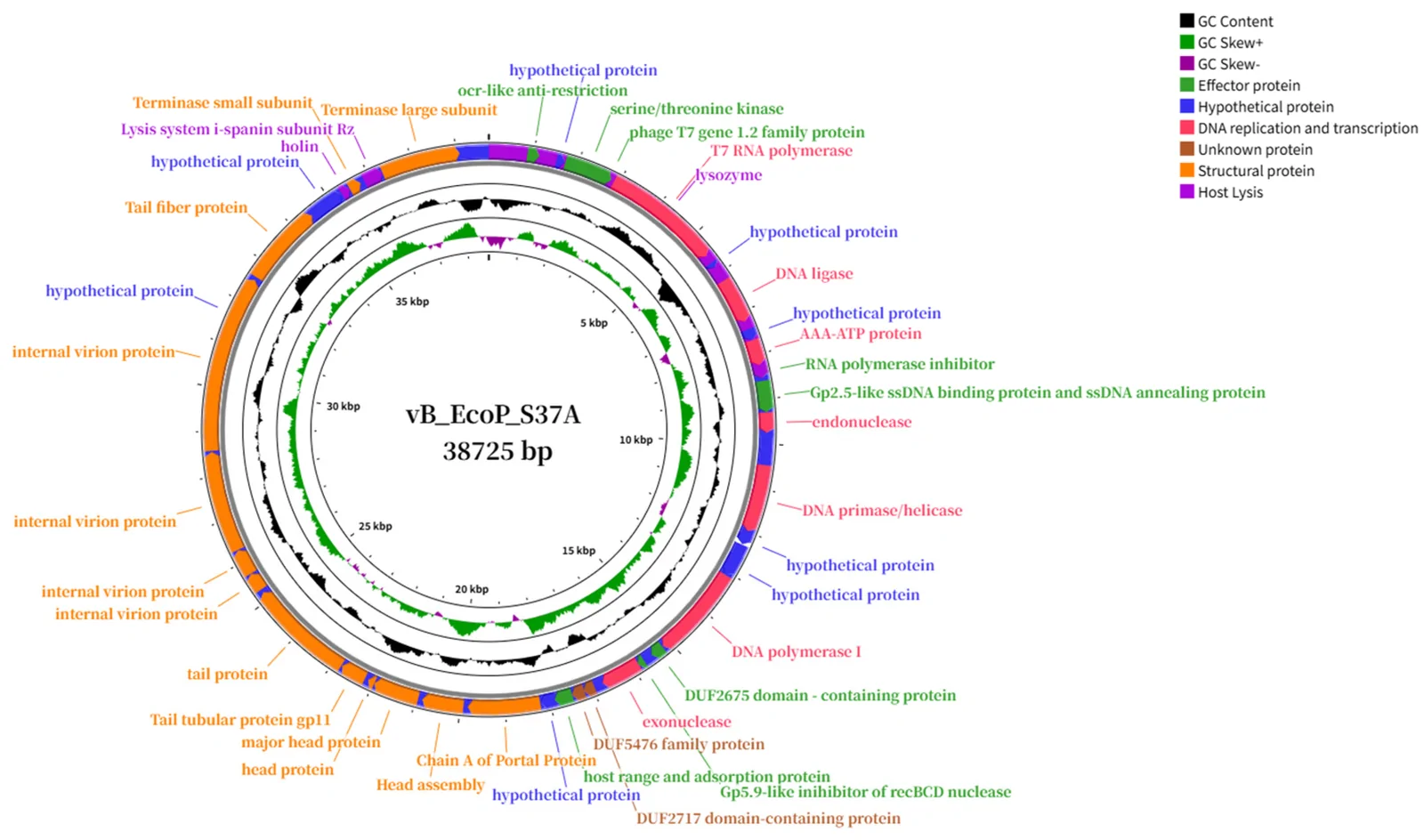
Circular genome maps of vB_EcoM_S37A. ORFs are color-coded by functional categories; tRNA genes are marked on the maps.

In contrast, phage vB_EcoP_S37A has a smaller genome, encoding 41 ORFs, of which 32 (78.04%) are known functional proteins and 9 (21.95%) are hypothetical proteins; no tRNA genes were predicted. Functional annotation assigned the ORFs into three modules: structural assembly proteins (13), lysis-related proteins (3), and DNA replication/transcription/repair proteins (16) (Figure 7). Detailed information on phage protein functions is provided in Table S3. Among the structural proteins, ORF28, ORF31, and ORF36 encode the major head protein, tail protein, and tail fibre protein, respectively; ORF35 encodes an internal virion protein containing an SLT-family transglycosylase domain at residues 16–128. Lysis-related proteins include lysozyme (ORF13), holin (ORF38), and i-spanin Rz subunit (ORF40). Among the DNA-related proteins, ORF39 and ORF41 encode the terminase small and large subunits, respectively.

### 3.10. Phylogenetic Analysis of vB_EcoM_S37

**Figure 8:**
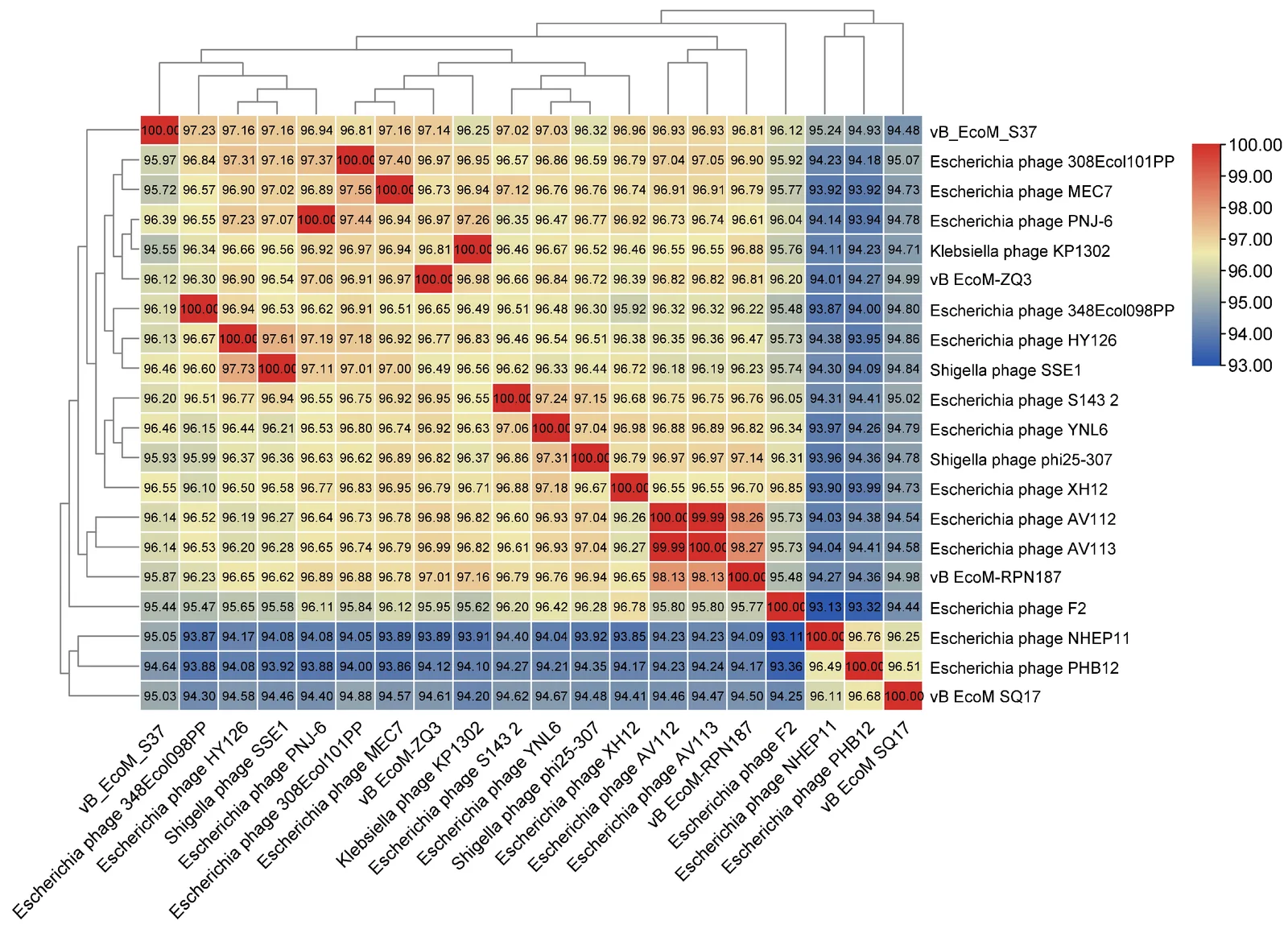
Average nucleotide identity (ANIb) heatmap of vB_EcoM_S37.

NCBI BLASTn results showed that vB_EcoM_S37 shares high sequence similarity with members of the genus *Mosigvirus* within the family *Straboviridae*, with the highest identity (98.24%, 92% coverage) to Escherichia phage YNL6 (PZ154896.1). ANIb analysis revealed that vB_EcoM_S37 shares ANIb values of 96.46%, 95.79%, 95.44%, and 95.03% with YNL6, Escherichia phage 308Ecol101PP, Escherichia phage F2, and vB_EcoM_SQ17, respectively, all exceeding the 95% species demarcation threshold, indicating that they belong to the same species (Figure 8).

**Figure 9:**
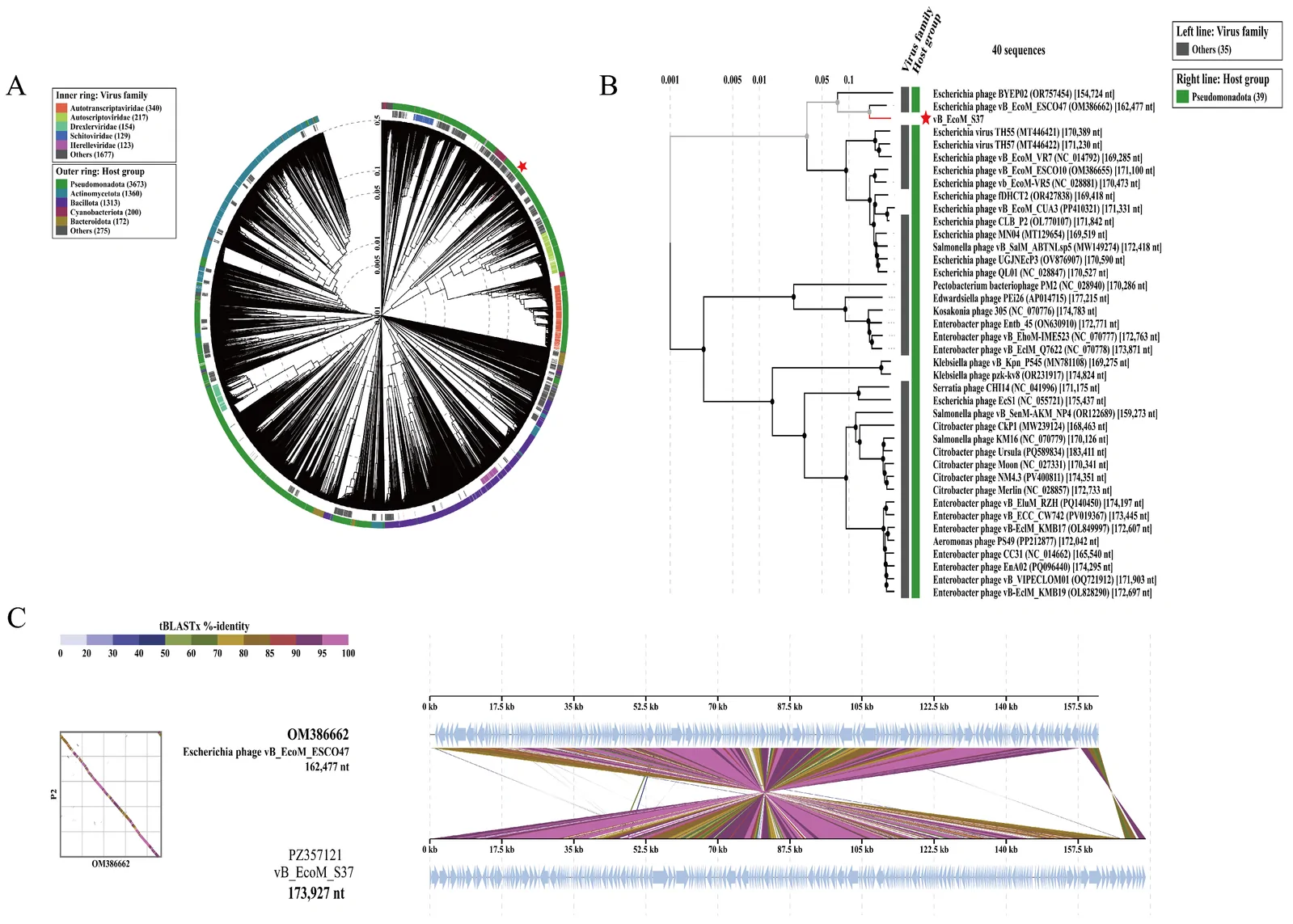
Phylogenetic analysis of vB_EcoM_S37. (**A**) Circular proteomic tree generated by ViPTree. (**B**) Rectangular proteomic tree. (**C**) Genome-to-genome comparison with Escherichia phage vB_EcoM_ESCO47. The red star indicates vB_EcoM_S37.

The ViPTree circular proteomic tree analysis revealed that vB_EcoM_S37 clustered with phages whose known hosts are predominantly within the phylum Pseudomonadota (Figure 9A). At the viral taxonomic level, the phage did not cluster with members of the family Straboviridae on the phylogenetic tree, but instead formed a distinct branch (Figure 9A). The rectangular proteomic tree further showed that vB_EcoM_S37 shares the same branch with phage vB_EcoM_ESCO47 (also an unclassified genus) (Figure 9B), suggesting a relatively close phylogenetic relationship between the two phages, and the genome-to-genome comparison supported this close relationship (Figure 9C).

**Figure 10:**
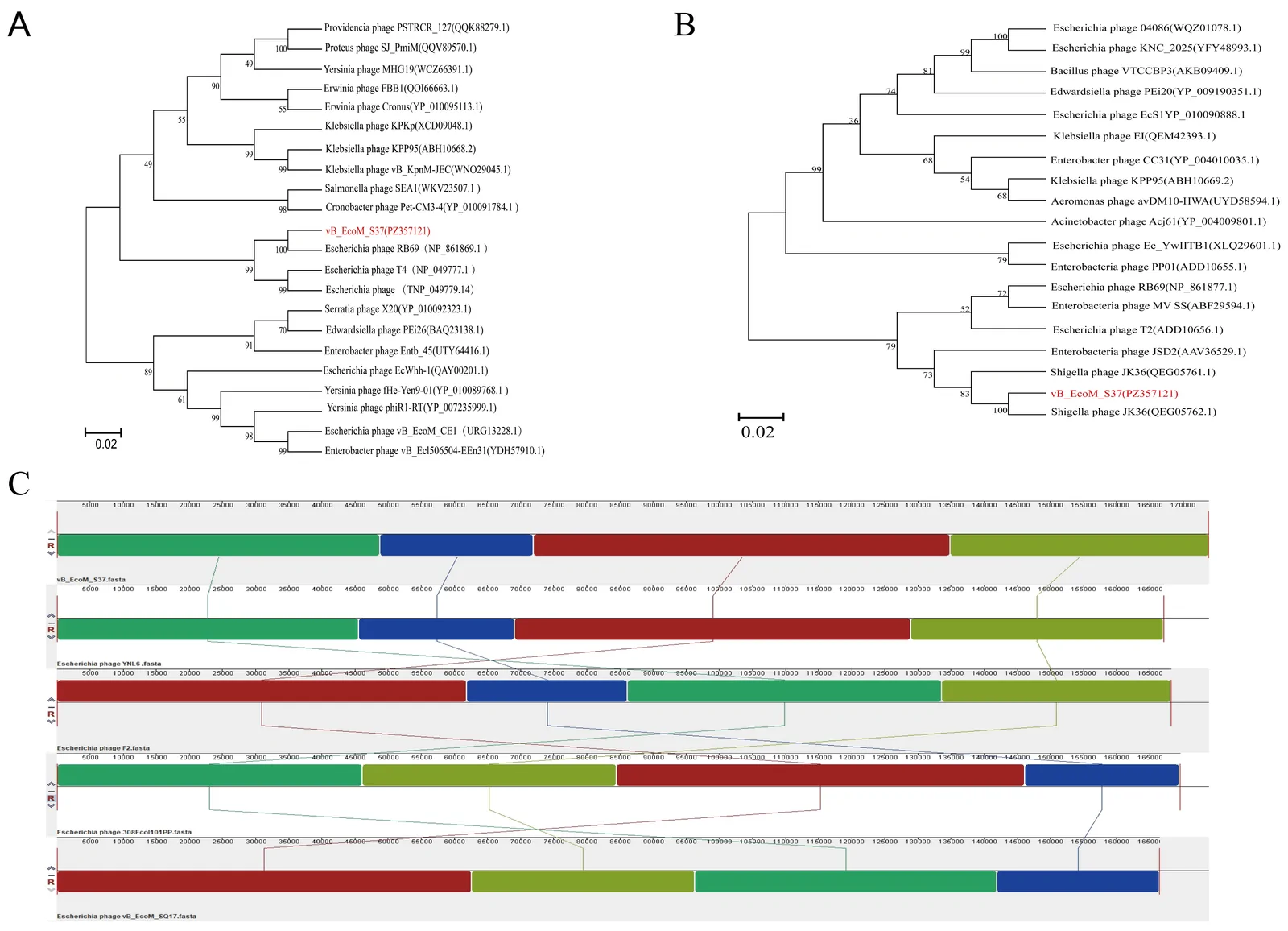
Genomic organization and phylogenetic analysis of vB_EcoM_S37. (**A**) Phylogenetic tree based on the amino acid sequence of the terminase large subunit (ORF2). (**B**) Phylogenetic tree based on major capsid protein (ORF10). (**C**) Mauve whole-genome alignment with four related phages. The red font indicates vB_EcoM_S37 and its GenBank accession number (PZ357121)

The terminase large subunit (ORF2)-based phylogenetic tree placed vB_EcoM_S37 within the same clade as phage RB69 (Figure 10A), whereas the major capsid protein (ORF10)-based tree clustered it with Shigella phage JK36 (Figure 10B). This incongruence suggests that the vB_EcoM_S37 genome may have undergone recombination, with different functional modules originating from distinct evolutionary lineages.

To investigate genomic rearrangements, vB_EcoM_S37 was compared with the above four related phages using Mauve whole-genome alignment. Four highly conserved locally collinear blocks (LCBs) were identified among the five genomes, indicating homologous regions maintained during evolution. The genome of vB_EcoM_S37 exhibited a high degree of synteny with that of YNL6, whereas it displayed complex multi-rearrangement patterns when compared with phages F2, 308Ecol101PP, and vB_EcoM_SQ17 (Figure 10C).

**Figure 11:**
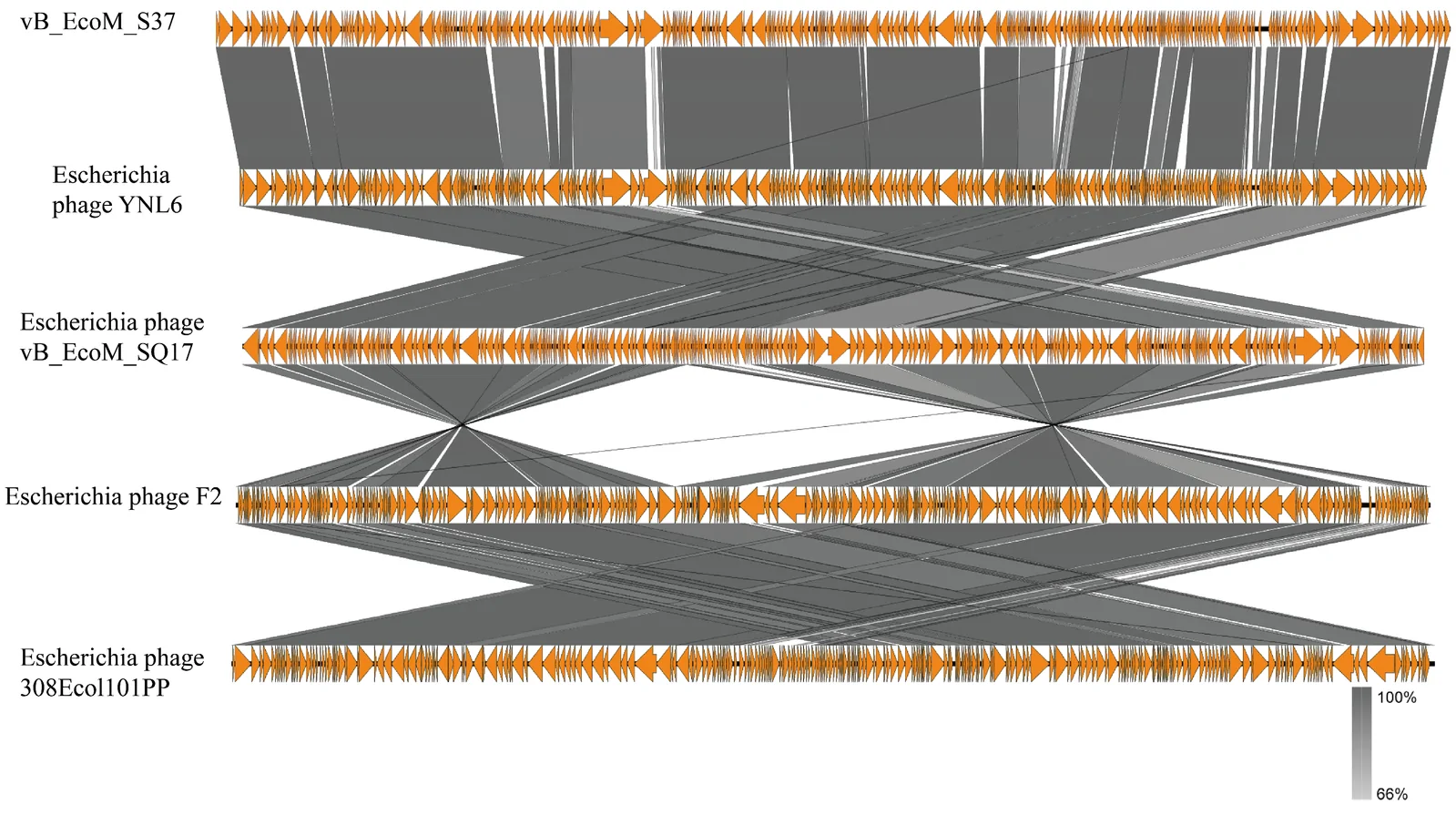
Reference-based synteny analysis using Easyfig with vB_EcoM_S37 as the reference. Although Mauve identified high synteny between vB_EcoM_S37 and YNL6, substantial gene-level differences exist between the two genomes.

To further analyze gene-level synteny and organizational differences, we performed a reference-based synteny analysis using Easyfig with vB_EcoM_S37 as the reference sequence (Figure 11). The analysis revealed that, although Mauve identified a high degree of synteny between vB_EcoM_S37 and YNL6, substantial gene-level variations exist between the two genomes.

**Figure 12:**
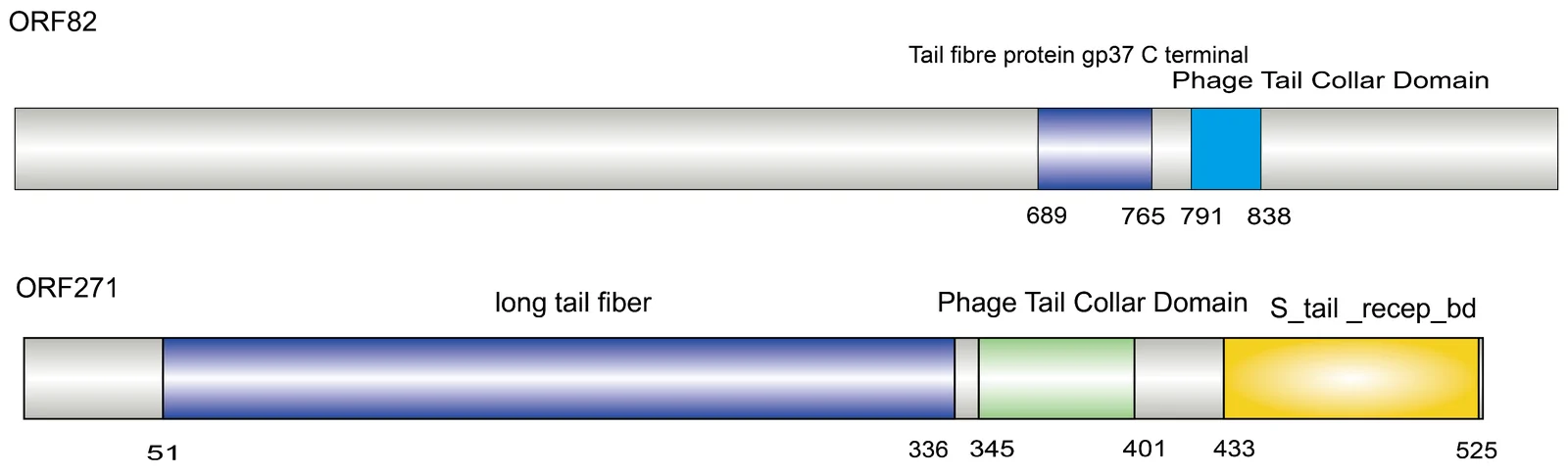
PhaRBP screening identified 12 putative RBPs in vB_EcoM_S37. ORF82 contains a gp37_C domain and shares significant homology with T4 gp37 (45.72% identity, 100% coverage, E = 0.0), indicating it is a true gp37 homolog. ORF271 harbors an S_tail_recep_bd domain but showed lower similarity (25.90% identity, 11% coverage, E = 6 × 10^−4^), suggesting it may mediate host adsorption via a mechanism distinct from gp37.

PhaRBP-based screening predicted 12 putative receptor-binding proteins in vB_EcoM_S37, encoded by ORF14, ORF27, ORF66, ORF78, ORF79, ORF80, ORF82, ORF157, ORF211, ORF265, ORF266, and ORF271. CD-Search analysis revealed that ORF82 contains a gp37_C domain (pfam12604), and ORF271 contains an S_tail_recep_bd domain (pfam14928). Sequence alignment of ORF82 and ORF271 with the T4 phage gp37 protein showed that ORF82 shares significant homology with T4 gp37, with 45.72% identity, 100% query coverage, and an E-value of 0.0, indicating that ORF82 is a true homolog of T4 gp37. In contrast, ORF271 showed 25.90% identity and 11% coverage with T4 gp37, with an E-value of 6 × 10^−4^, suggesting that ORF271 may mediate host adsorption through a recognition mechanism distinct from that of gp37 (Figure 12).

### 3.11. Phylogenetic Analysis of vB_EcoP_S37A

**Figure 13:**
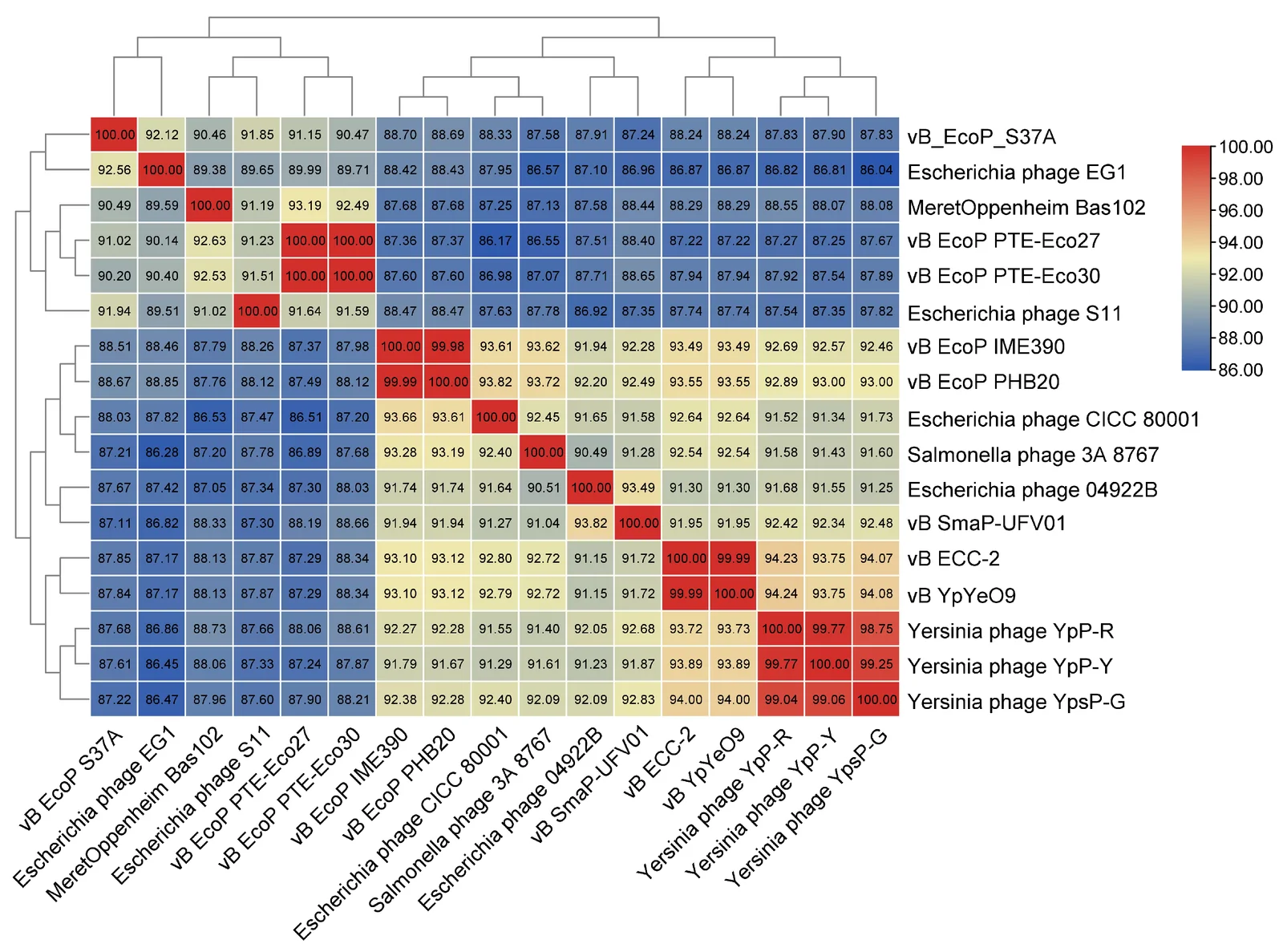
Average nucleotide identity (ANIb) heatmap of vB_EcoP_S37A.

NCBI BLASTn results indicated that vB_EcoP_S37A is closely related to members of the genus *Teseptimavirus* within the family *Autotranscriptaviridae*, but with identities all below 95%; the highest identity (94.45%, 83% coverage) was with Escherichia phage 04922B (OP331230.1, Xiamen, China). ANIb analysis further revealed that vB_EcoP_S37A shares ANIb values below 95% with all related phages, with the highest being 92.56% (against Escherichia phage EG1), confirming that vB_EcoP_S37A represents a novel species within the genus *Teseptimavirus* according to current ICTV criteria (ANI < 95% for species demarcation) [20] (Figure 13).

**Figure 14:**
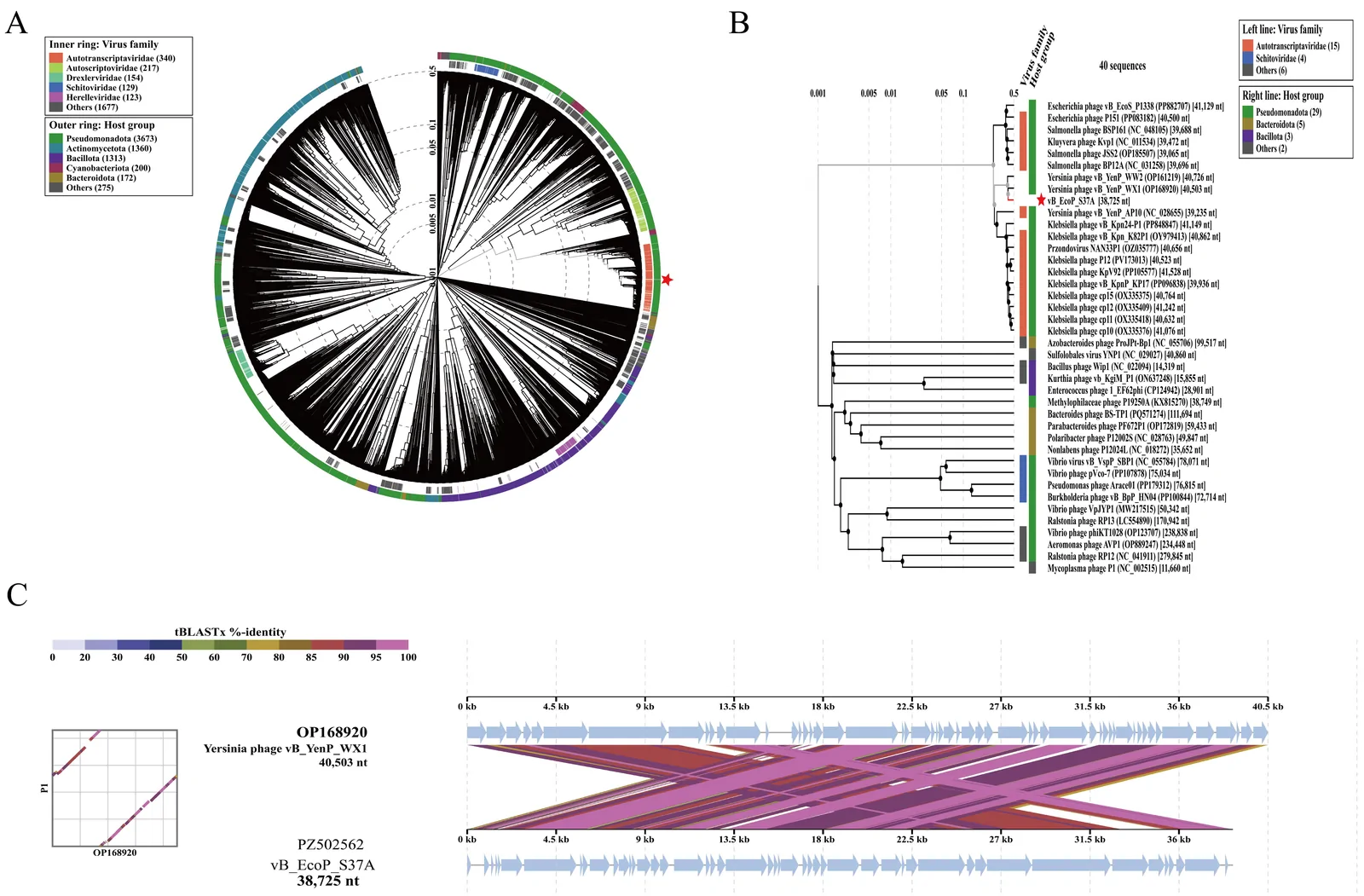
Phylogenetic analysis of vB_EcoP_S37A. (**A**) Circular proteomic tree generated by ViPTree. (**B**) Rectangular proteomic tree. (**C**) Genome-to-genome comparison with Yersinia phage vB_YenP_WX1. The red star indicates vB_EcoP_S37A.

ViPTree proteomic analysis placed vB_EcoP_S37A within the family Autotranscriptaviridae (Figure 14A). The rectangular proteomic tree showed that vB_EcoP_S37A is most closely related to Yersinia phage vB_YenP_WX1, within the genus *Teseptimavirus* (Figure 14B), and the genome-to-genome comparison supported this close relationship (Figure 14C).

**Figure 15:**
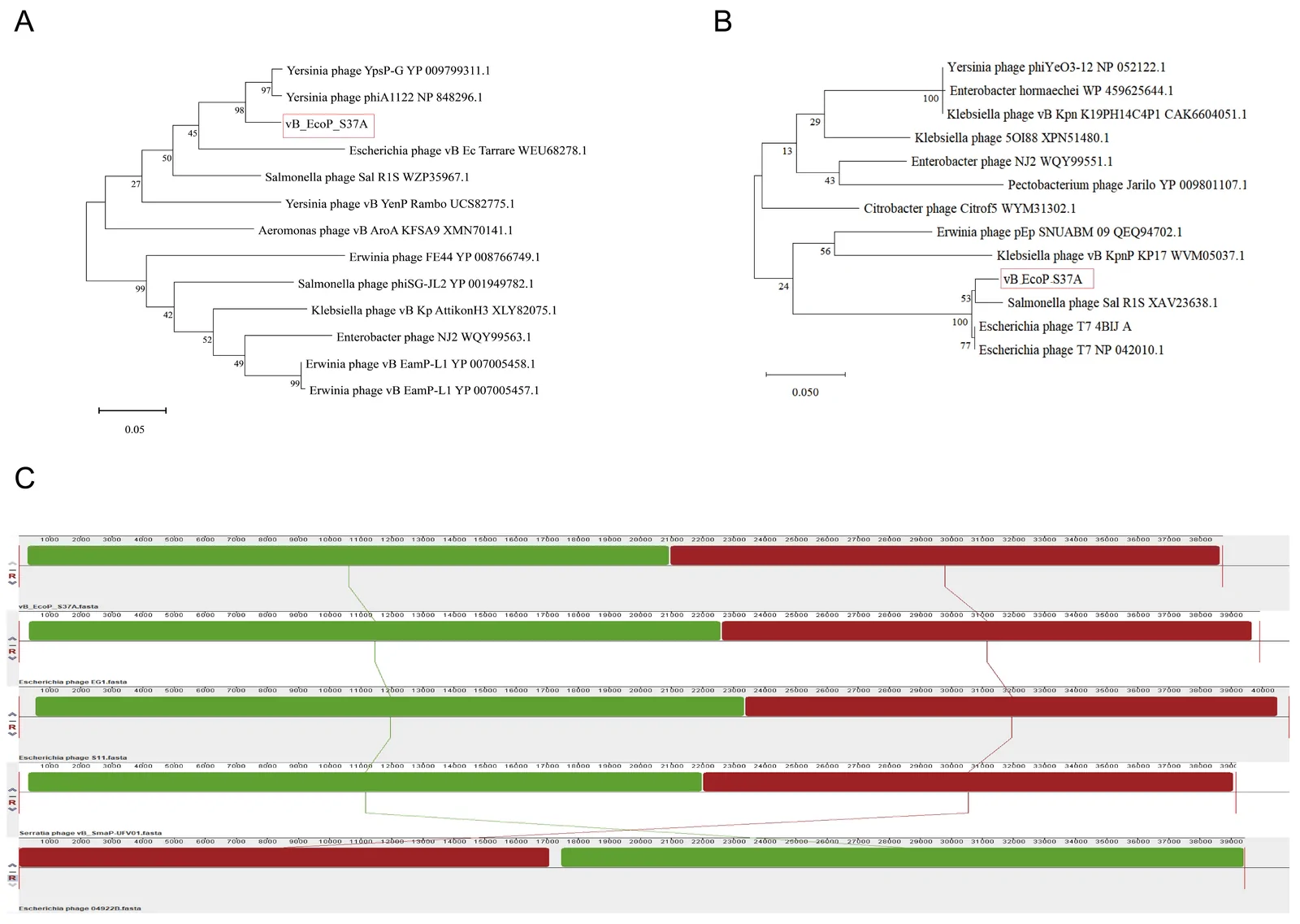
Genomic organization and phylogenetic analysis of vB_EcoP_S37A. (**A**) Phylogenetic tree based on major capsid protein (ORF28). (**B**) Phylogenetic tree based on terminase large subunit (ORF41). (**C**) Mauve whole-genome alignment with related phages.

Phylogenetic trees were constructed based on the major capsid protein (ORF28) and the large subunit of terminase (ORF41). The major capsid protein tree showed that vB_EcoP_S37A did not cluster with any known phage but formed an independent clade (Figure 15A), indicating significant divergence in its major capsid protein sequence from reported phages. The terminase large subunit tree placed vB_EcoP_S37A in a small clade with Salmonella phage Sal RlS (Figure 15B), further supporting its classification as a novel phage.

To further analyze its genomic structure, vB_EcoP_S37A was compared with EG1, 04922B, S11 (PQ821741.1), and Serratia phage vB_SmaP-UFV01 (NC_047895.1) using Mauve whole-genome alignment (Figure 15C). All four phage genomes contained two complete LCBs, all in the forward orientation, with no inversions detected, indicating that the genome of vB_EcoP_S37A is structurally conserved. Regarding LCB order, vB_EcoP_S37A shared the same order as EG1, S11, and vB_SmaP-UFV01, while 04922B exhibited a reversed order.

**Figure 16:**
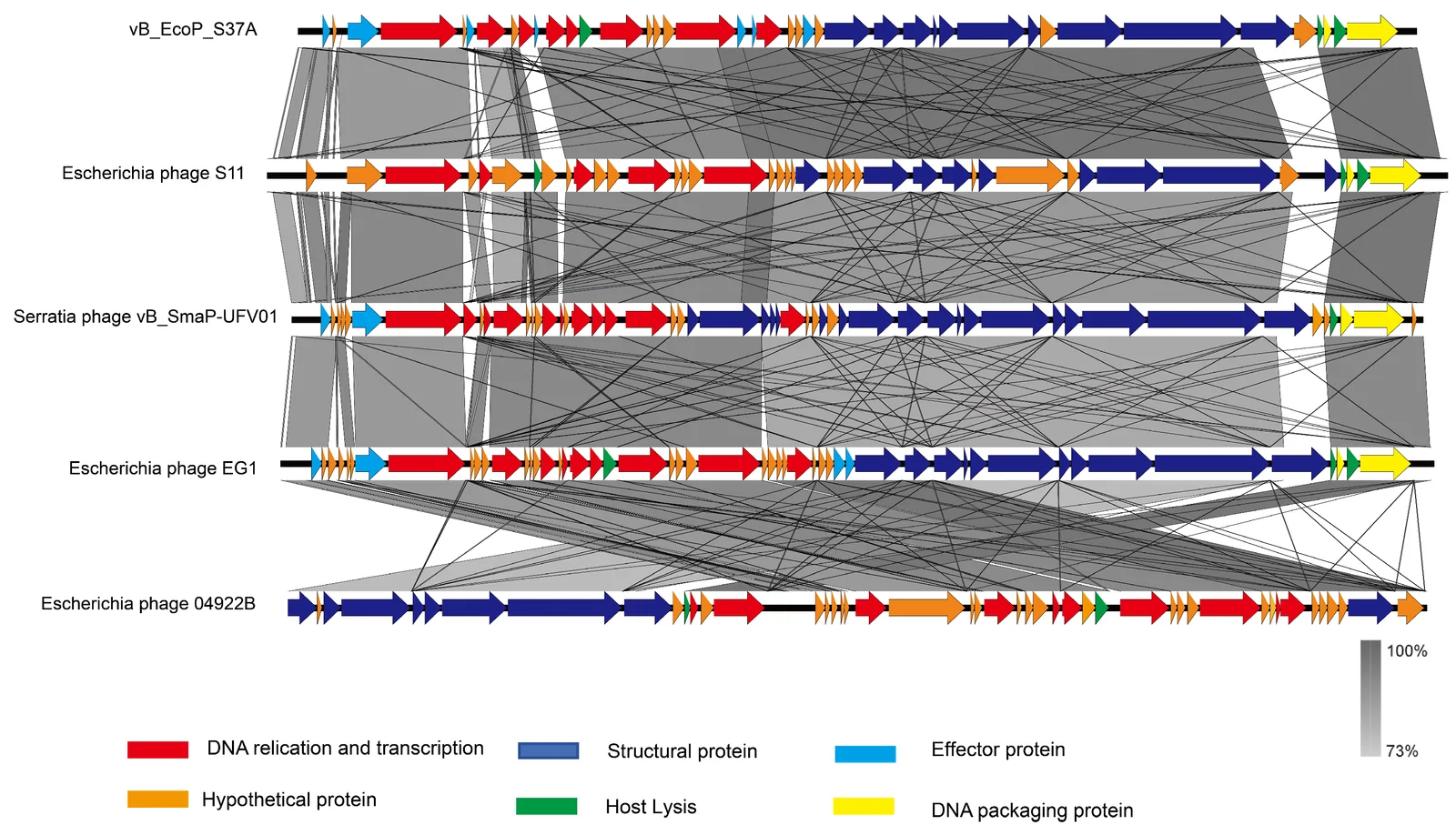
Synteny analysis of vB_EcoP_S37A and related phages. vB_EcoP_S37A, S11, Serratia phage vB_SmaP-UFV01, and EG1 share conserved gene arrangements, with functional modules (DNA replication/transcription, structural proteins, lysis, and DNA packaging) maintaining consistent syntenic order. In contrast, Escherichia phage 04922B exhibited large-scale genomic inversions.

Synteny analysis showed that vB_EcoP_S37A, S11, Serratia phage vB_SmaP-UFV01, and EG1 shared conserved gene arrangements, with functional modules including DNA replication and transcription, structural proteins, lysis, and DNA packaging maintaining consistent syntenic order. In contrast, Escherichia phage 04922B exhibited large-scale genomic inversions (Figure 16).

### 3.12. Structural Analysis of vB_EcoP_S37A Tail Fibre Protein

**Figure 17:**
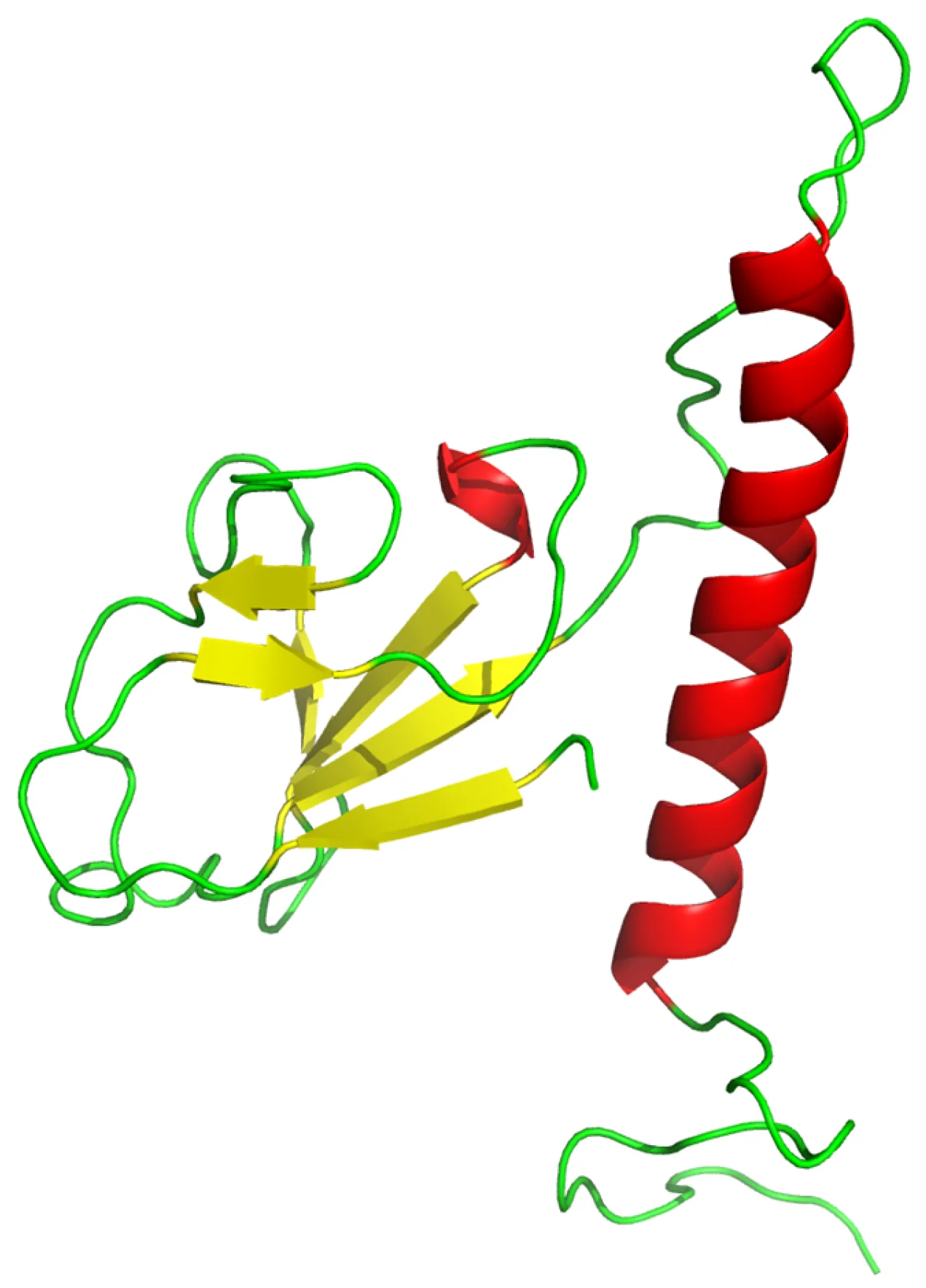
Predicted three-dimensional structure of ORF36 generated by Phyre2 homology modelling. The model is colored by secondary structure: red, α-helices; yellow, β-sheets; green, flexible loop/coil regions. Model dimensions: 53.3 × 33.6 × 60.3 Å.

The halo phenotype observed around plaques of vB_EcoP_S37A prompted us to investigate whether the phage encodes a depolymerase. However, genomic annotation revealed that ORF36 encodes a tail fibre protein. To gain structural insights, ORF36 was subjected to homology modelling and tertiary structure prediction using the Phyre2 server. The predicted model reveals an elongated two-domain conformation: the N-terminal region is predominantly composed of β-strands (50%), while the C-terminal region contains a long α-helical bundle (5%), with the two domains connected by a flexible random coil region (2%). This high proportion of random coil contributes to structural flexibility. The C-terminal α-helical bundle may facilitate tail fibre extension and trimerization, whereas the N-terminal β-strand-rich region is hypothesized to be involved in host receptor recognition and binding (Figure S3 and Figure 17). These structural features are consistent with a typical phage tail fibre protein architecture.

**Figure 18:**
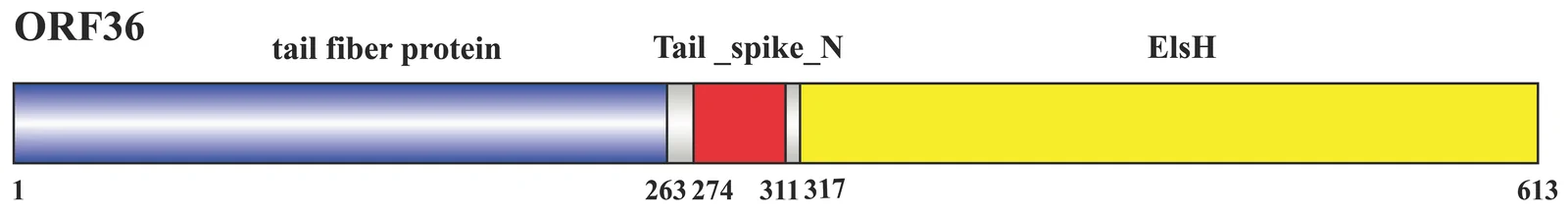
PhaRBP screening identified five putative RBPs in vB_EcoP_S37A (ORF28, ORF31, ORF35, ORF36, and ORF37). ORF36 contains three domains: tail fiber protein (1–263), Tail_spike_N (274–311), and ElsH (317–613). Its N-terminal region (1–173) shares 82.95% identity with phage gp17, while the C-terminal region shows no similarity, suggesting a unique receptor recognition function.

PhaRBP-based screening predicted five putative receptor-binding proteins in vB_EcoP_S37A, encoded by ORF28, ORF31, ORF35, ORF36, and ORF37. CD-Search domain analysis (Figure 18) showed that ORF36 consists of three domains: tail fiber protein (residues 1–263), Tail_spike_N (residues 274–311), and ElsH (residues 317–613). Further BLASTp alignment of ORF36 with phage gp17 protein revealed that the N-terminal region of ORF36 (residues 1–173) is highly conserved with the N-terminus of gp17 (82.95% identity), while no similarity was detected in the C-terminal regions, suggesting that the C-terminus of ORF36 may possess a unique receptor recognition function.

## 4. Discussion

In this study, two lytic phages targeting swine-derived ExPEC, vB_EcoM_S37 and vB_EcoP_S37A, were isolated from swine farm wastewater. Although both phages utilize *E. coli* S37 as their host, they differ markedly in genome architecture, biological characteristics, and evolutionary strategies, representing two distinct survival strategies among phages. Transmission electron microscopy revealed that vB_EcoM_S37 exhibits a myovirus morphology, while vB_EcoP_S37A displays a podovirus morphology. Both phages were able to infect *Escherichia coli* strains derived from different animal hosts, suggesting their potential value in cross-host infection control. Compared with previously isolated phages vB_Eco4M-7 [30] and KFS-EC3 [31], vB_EcoM_S37 and vB_EcoP_S37A exhibited shorter latent periods and larger burst sizes, suggesting stronger infectivity and replication efficiency.

Bacteriophages are composed of nucleic acids and proteins, and their activity can be affected by high temperatures, as well as overly acidic or alkaline environments [32]. In this study, vB_EcoM_S37 and vB_EcoP_S37A remained stable at temperatures ranging from 40 to 60 °C and across a pH range of 4–11. Compared with phages SKA49 and SKA64 isolated by Sattar et al. [33], vB_EcoM_S37 and vB_EcoP_S37A exhibited superior thermal and pH stability. For bacteriophage biologics intended for clinical therapy, it is essential to examine their genomes for the presence of any virulence or antibiotic resistance genes [34]. Therefore, comprehensive genomic analysis of the two phages was performed, and the results showed that neither vB_EcoM_S37 nor vB_EcoP_S37A carried relevant resistance or virulence genes, supporting their safety profile for potential application.

The genomic size difference is particularly striking—vB_EcoM_S37 (173,927 bp) is 4.5-fold larger than vB_EcoP_S37A (38,725 bp). This difference is directly reflected in their coding capacities: vB_EcoM_S37 encodes 276 ORFs, whereas vB_EcoP_S37A encodes only 41. At the functional level, vB_EcoM_S37 possesses significantly more genes involved in DNA metabolism and replication (76 vs. 16) and lysis (5 vs. 3), which together are associated with a shorter latent period (5 min vs. 10 min), a larger burst size (1173 vs. 210 PFU/cell), and more sustained in vitro antibacterial activity (12 h vs. 8 h). The substantially larger burst size of vB_EcoM_S37, together with its greater number of lysis-associated genes, suggests a potential link between the additional lysis proteins and more efficient progeny release. Additionally, vB_EcoM_S37 carries two tRNA genes, indicating greater translational autonomy [35], whereas vB_EcoP_S37A relies entirely on the host translation machinery. This contrast between “heavy-equipped” and “lightweight” strategies reflects the distinct evolutionary adaptations employed by the two phages in response to the same host environment.

The difference in host range can also be explained at the genomic level. vB_EcoM_S37 encodes a tail fibre protein (ORF 271) and other structural recognition proteins, enabling it to recognize 43.55% of tested strains, whereas vB_EcoP_S37A, although also encoding a tail fibre protein (ORF36), exhibits limited recognition capacity, lysing only 6.45% of strains. Notably, vB_EcoP_S37A forms plaques with halos, and its internal virion protein (ORF35) contains an SLT-family transglycosylase domain—a feature highly similar to the T7 gp16 protein, which facilitates phage genome translocation across the host peptidoglycan layer during infection [36,37]. It is tempting to speculate that this domain may contribute to the halo phenotype; however, this remains a hypothesis that requires experimental validation through enzymatic assays, protein expression, and mutational analysis. The tail fibre complex (gp36/gp37) may also be involved in halo formation, and its role warrants further investigation.

At the evolutionary level, the two phages exhibit contrasting patterns. The diversity of LCB arrangements and complex multi-rearrangement patterns in the vB_EcoM_S37 genome are suggestive of multiple recombination events—a key driver of phage evolution that may have contributed to its broader host adaptability [38]. In contrast, vB_EcoP_S37A displays a highly conserved genome structure, with no inversions or rearrangements detected, and its LCB order is consistent with that of closely related phages such as EG1 [39] and S11. This conservation may indicate that its core functions are already highly optimized, and any structural variation could compromise its infection efficiency.

The taxonomic status of vB_EcoP_S37A is particularly noteworthy—ANIb analysis showed that its similarity to all known *Teseptimavirus* members is below 95%, establishing it as a novel species within this genus [40]. The independent branch formed by vB_EcoP_S37A in the major capsid protein phylogeny further supports this conclusion. This finding enriches the species diversity of the family *Autotranscriptaviridae* and provides a reference genome for future taxonomic studies within this genus.

Importantly, the two phages exhibit highly complementary biological characteristics. vB_EcoM_S37 has a broad host range but showed bacterial regrowth after 8 h, suggesting potential host escape, while vB_EcoP_S37A has a narrow host range but produces halo plaques. Although halo phenotypes in other phages have been linked to depolymerase activity that degrades capsular polysaccharides or lipopolysaccharides on the host surface [41], it remains to be investigated whether vB_EcoP_S37A produces such an enzyme. We hypothesize that the combined use of both phages may produce a synergistic effect: such enzymatic activity from vB_EcoP_S37A degrades surface polysaccharides, exposing more receptor sites and enhancing vB_EcoM_S37 adsorption; meanwhile, the two phages target different receptors on the same host, reducing the risk of dual escape through single-receptor mutation. This hypothesized synergy provides a theoretical foundation for the development of phage cocktail formulations but requires experimental validation in future studies.

Nevertheless, this study has certain limitations. All experiments were conducted in vitro, and the safety and efficacy of both phages in animals remain to be verified. Additionally, the mechanism underlying the halo phenotype of vB_EcoP_S37A, the specific function of the SLT domain, and the potential synergistic effect between the two phages require further experimental validation. Future studies should evaluate the therapeutic efficacy of each phage alone and in combination using swine ExPEC infection models, and characterize the depolymerase activity, the frequency of resistant mutant emergence, and its synergistic mechanism.

## 5. Conclusions

Two lytic bacteriophages, vB_EcoM_S37 and vB_EcoP_S37A, targeting swine-derived extraintestinal pathogenic *E. coli*, were successfully isolated from swine farm wastewater. vB_EcoM_S37 belongs to the genus *Mosigvirus* (family *Straboviridae*), with a 173,927-bp genome carrying two tRNAs, and exhibits a short latent period (5 min), large burst size (1173 PFU/cell), broad host range (43.55%), and evidence of multiple recombination events. vB_EcoP_S37A represents a novel species within the genus *Teseptimavirus* (family *Autotranscriptaviridae*; ANIb < 95%), with a 38,725-bp structurally conserved genome. Structural analysis of its tail fibre protein (ORF36) revealed an elongated two-domain conformation consistent with a typical phage tail fibre architecture; additionally, its internal virion protein (ORF35) contains an SLT-family transglycosylase domain, which is hypothesized to be associated with the halo phenotype, although this requires experimental confirmation. Both phages display favourable thermal and pH stability and carry no antibiotic resistance or virulence genes. The high complementarity between the two phages in host range, genomic architecture, and evolutionary strategy provides a theoretical basis and candidate strains for the development of phage cocktail formulations, although their therapeutic efficacy remains to be evaluated in vivo.

## Figures and Tables

**Table 1:** Host range of phages vB_EcoM_S37 and vB_EcoP_S37A.

Strain	Source	Lysis by vB_EcoM_S37	Lysis by vB_EcoP_S37A	Strain	Source	Lysis vB_EcoM_S37	Lysis by vB_EcoP_S37A
*E. coli* S1	Swine	−	−	*E. coli* S36	Swine	−	−
*E. coli* S2	Swine	−	−	*E. coli* S37	Swine	+	+
*E. coli* S3	Swine	−	−	*E. coli* S38	Swine	−	−
*E. coli* S4	Swine	−	−	*E. coli* S39	Swine	−	−
*E. coli* S5	Swine	−	+	*E. coli* S40	Swine	−	−
*E. coli* S6	Swine	−	−	*E. coli* C1	Chicken	−	−
*E. coli* S7	Swine	−	−	*E. coli* C2	Chicken	−	−
*E. coli* S8	Swine	−	−	*E. coli* C3	Chicken	−	−
*E. coli* S9	Swine	−	−	*E. coli* C4	Chicken	+	−
*E. coli* S10	Swine	−	−	*E. coli* C5	Chicken	−	−
*E. coli* S11	Swine	+	−	*E. coli* C6	Chicken	+	−
*E. coli* S12	Swine	−	+	*E. coli* C7	Chicken	+	−
*E. coli* S13	Swine	−	−	*E. coli* C8	Chicken	−	−
*E. coli* S14	Swine	−	−	*E. coli* C9	Chicken	+	−
*E. coli* S15	Swine	−	−	*E. coli* C10	Chicken	+	−
*E. coli* S16	Swine	+	−	*E. coli* R1	Rabbit	+	−
*E. coli* S17	Swine	+	−	*E. coli* R2	Rabbit	+	−
*E. coli* S18	Swine	−	−	*E. coli* R3	Rabbit	+	−
*E. coli* S19	Swine	+	−	*E. coli* R4	Rabbit	+	−
*E. coli* S20	Swine	−	+	*E. coli* R5	Rabbit	+	−
*E. coli* S21	Swine	−	−	*E. coli* R6	Rabbit	+	−
*E. coli* S22	Swine	−	−	*E. coli* R7	Rabbit	−	−
*E. coli* S23	Swine	+	−	*E. coli* P1	Peacock	+	−
*E. coli* S24	Swine	+	−	*E. coli* P2	Peacock	+	−
*E. coli* S25	Swine	−	−	*E. coli* P3	Peacock	−	−
*E. coli* S26	Swine	+	−	*E. coli* P4	Peacock	+	−
*E. coli* S27	Swine	−	−	*E. coli* P5	Peacock	−	−
*E. coli* S28	Swine	−	−	*P.mirabilis *S1	Swine	−	−
*E. coli* S29	Swine	−	−	*P.mirabilis S2*	Swine	−	−
*E. coli* S30	Swine	−	−	*K.pneumoniae *S1	Swine	−	−
*E. coli* S31	Swine	−	−	*K.pneumoniae *S2	Swine	−	−
*E. coli* S32	Swine	−	−	*K.pneumoniae *S3	Swine	−	−
*E. coli* S33	Swine	−	−	*K.pneumoniae *S4	Swine	−	−
*E. coli* S34	Swine	−	−	*K.pneumoniae *S5	Swine	−	−
*E. coli* S35	Swine	−	−				

Note: +, lytic; −, no lysis. Phage vB_EcoM_S37 lysed 27/62 *E. coli* strains (43.55%); phage vB_EcoP_S37A lysed 4/62 *E. coli* strains (6.45%). Neither phage lysed *K. pneumoniae* or *P. mirabilis*.

## Data Availability

The original contributions presented in this study are included in the article/Supplementary Material. Further inquiries can be directed to the corresponding authors.

## Supplementary Materials

The following are available online at https://www.mdpi.com/article/10.3390/vetsci13090943/s1.

## Abbreviations

The following abbreviations are used in this manuscript:

*E. coli*	*Escherichia coli*
*K. pneumoniae*	*Klebsiella pneumoniae*
*P. mirabilis*	*Proteus mirabilis*
MOI	The optimal multiplicity of infection
phage	bacteriophage
