# Supplementary material for: Identification and Genomic Characterization of a Novel Teseptimavirus Species (vB_EcoP_S37A) and a Mosigvirus Phage (vB_EcoM_S37) Infecting Swine Extraintestinal Pathogenic Escherichia coli
Source: Vet Sci. 2026 Sep 10;13(9):943. doi: 10.3390/vetsci13090943 (PMC13611991; doi:10.3390/vetsci13090943)
Supplement: Supplementary file 1 [file vetsci-13-00943-s001.zip › vetsci-4491518-supplementary.pdf]

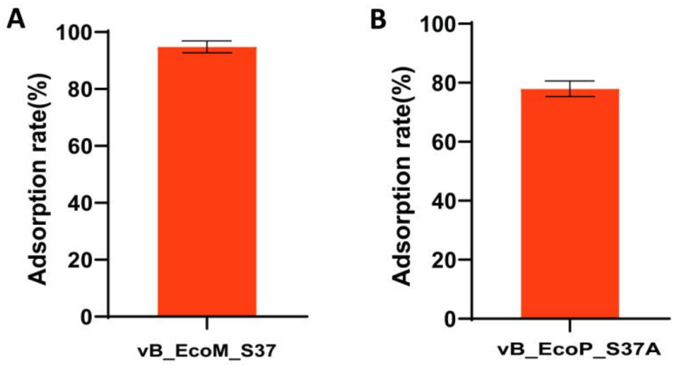

Figure. S1. (A) Adsorption rate of phage vB_EcoM_S37. **(B)** Adsorption rate of phage vB_EcoP_S37A.

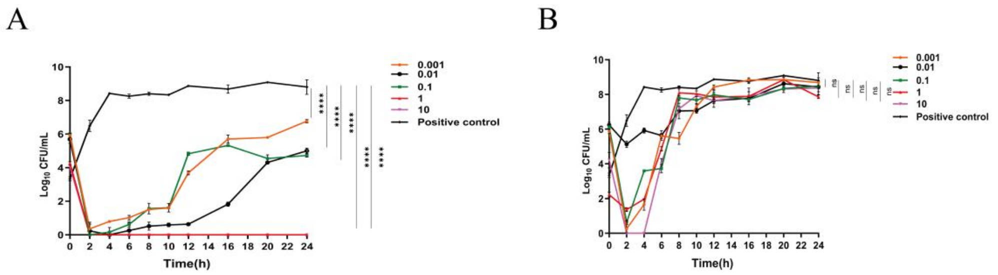

Figure. S2. (A) In vitro colony count assay of vB_EcoM_S37.(B)In vitro colony count assay of vB_EcoP_S37A.

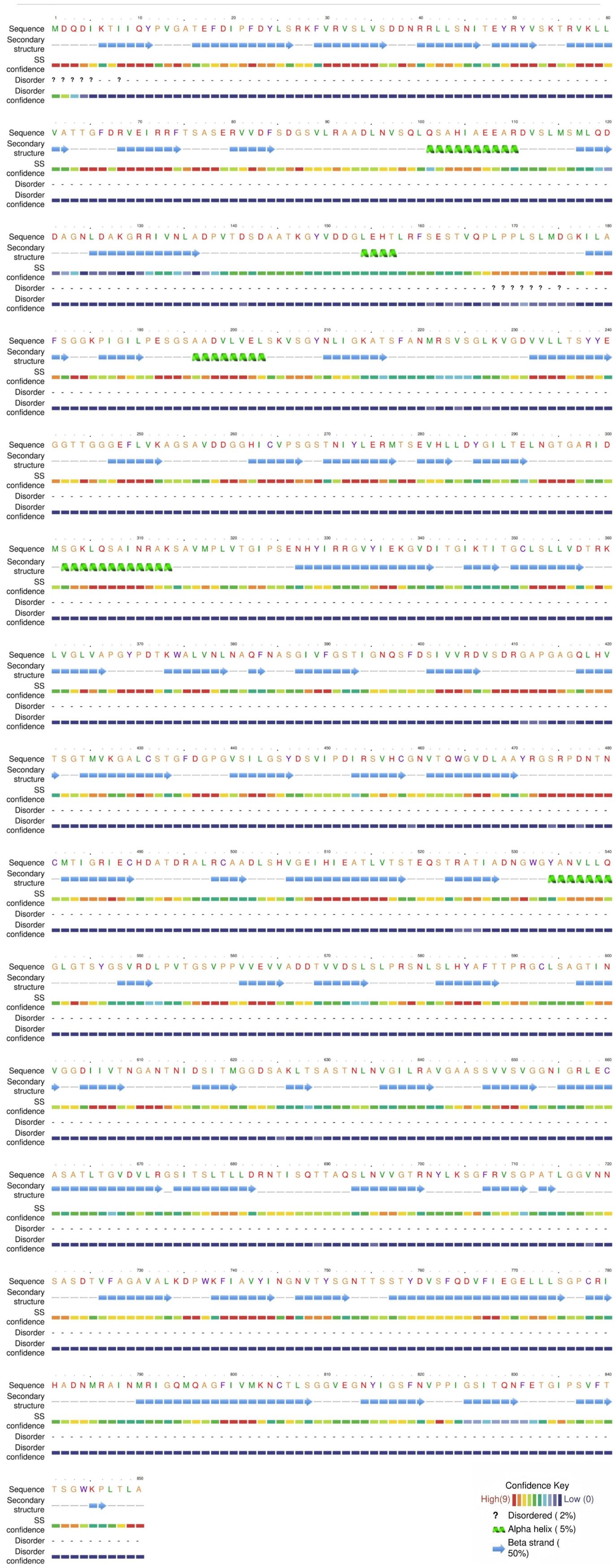

Figure. S3. Secondary structure composition analysis of the tail fiber protein (ORF36). Analysis of the

predicted structure reveals a distinct secondary structure profile, characterized by the highest proportion of random coils (2%), followed by β-sheets (50%), and α-helices (5%). These structural features are consistent with the typical morphology of phage tail fiber proteins.

Table S1

Prediction of phage lifestyle and genomic safety assessment of vB_EcoM_S37andvB_EcoP_S37A.

Tools	Phage lifestyle	Genomic Safety of Phages			
		Genomic mobility elements	Integron elements	Antibiotic resistance genes	Virulence factors
PhageAI	Virulent				
PhaTYP (score)	Lytic (1)				
Integron Finder			None		
CONJscan		None			
CARD				None	
VFDB					None

Table S2

ORF	Product	Source of Function	Accession	Functional category
1	terminase small subunit	Escherichia phage RB69	NP_861868.1	DNA packaging protein
2	terminase large subunit	Escherichia phage RB69	NP_861869.1	DNA packaging protein
3	tail sheath	Escherichia phage RB69	NP_861870.1	Structural protein
4	tail protein	Escherichia phage RB69	NP_861871.1	Structural protein
5	portal protein	Escherichia phage RB69	NP_861872.1	Structural protein
6	hypothetical protein	Escherichia phage vB_EcoM	UPW39477.1	Hypothetical protein
7	hypothetical protein	Klebsiella phage vB_Kpn_AM_K6	WWS25200.1	Hypothetical protein
8	prohead assembly (scaffolding) protein	Escherichia phage vB_EcoP_EP32B	WPK28957.1	Assembly accessory protein
9	capsid assembly scaffolding protein, partial	Escherichia phage PNJ-6	WBM23415.1	Assembly accessory protein
10	major capsid protein	Shigella phage JK36	QEG05762.1	Structural protein
11	homing endonuclease	Enterobacteria phage ATK48	ANZ51368.1	Nucleic acid metabolism
12	hypothetical protein	Escherichia phage KNC_2025	YFY48992.1	Hypothetical protein
13	capsid vertex protein	Escherichia phage RB69	NP_861880.1	Structural protein
14	RNA ligase	Escherichia phage SF-E1	YEN86529.1	Nucleic acid metabolism
15	hypothetical protein	Escherichia phage RB69	NP_861882.1	Hypothetical protein
16	hypothetical protein	Escherichia phage RB69	NP_861883.1	Hypothetical protein
17	Hoc-like head decoration	Escherichia phage vB_EcoM_OE5505	YP_010072468.1	Structural protein
18	minor head protein inhibitor of protease	Escherichia phage RB69	NP_861885.1	Structural protein
19	DNA helicase	Escherichia phage RB69	NP_861886.1	Nucleic acid metabolism
20	DNA helicase	Escherichia phage RB69	NP_861887.1	Nucleic acid metabolism
21	hypothetical protein	Escherichia phage vB_Eco	QBQ76944.1	Hypothetical protein
22	hypothetical protein	Escherichia phage RB69	NP_861889.1	Hypothetical protein
23	UvsY-like recombination mediator	Escherichia phage RB69	NP_861890.1	Nucleic acid metabolism
24	baseplate wedge subunit	Citrobacter phage Moon	YP_009146639.1	Structural protein
25	baseplate hub	Escherichia phage T4	NP_813811.1	Structural protein
26	baseplate hub assembly protein	Escherichia phage RB69	NP_861893.1	Structural protein
27	baseplate hub	Escherichia phage RB69	NP_861894.1	Structural protein
28	baseplate hub assembly protein	Escherichia phage PTK	WP_015971592.1	Structural protein
29	baseplate hub subunit and tail length	Escherichia phage phiC120]	YP_010090031.1	Structural protein
30	baseplate tail tube cap	Escherichia phage RB69	NP_861897.1	Structural protein
31	tail tube	Escherichia phage RB69	ELS1646840.1	Structural protein
32	hypothetical protein	Escherichia phage AV113	WPK34673.1	Hypothetical protein
33	Alt-like RNA polymerase ADP-ribosyltransferase	Escherichia phage RB69	NP_861900.1	Nucleic acid metabolism
34	hypothetical protein	Escherichia phage RB69	NP_861901.1	Hypothetical protein
35	hypothetical protein	Escherichia phage vB_EcoP_EP32B	WPK28929.1	Hypothetical protein
36	hypothetical protein	Escherichia phage vB_Eco_NicPhage	CAJ1523397.1	Hypothetical protein
37	hypothetical protein	Escherichia coli	ELS1646871.1	Hypothetical protein
38	hypothetical protein	Shigella phage SP21535	XPN41008.1	Hypothetical

ORF	Product	Source of Function	Accession	Functional category
				protein
39	hypothetical protein	Escherichia phage OLB35	AYR04121.1	Hypothetical protein
40	hypothetical protein	Escherichia coli	WP_016058890.1	Hypothetical protein
41	hypothetical protein	Shigella phage PSD9	QXV72301.1	Hypothetical protein
42	hypothetical protein	Escherichia phage RB69	NP_861908.1	Hypothetical protein
43	hypothetical protein	Escherichia phage RB69	NP_861909.1	Hypothetical protein
44	hypothetical protein	Escherichia phage HX01	YP_010247106.1	Hypothetical protein
45	rIII lysis inhibitor accessory	Escherichia phage vB_EcoP_EP32B	WPK28918.1	Lysis-associated protein
46	hypothetical protein	Salmonella enterica	EBJ7676751.1	Hypothetical protein
47	hypothetical protein	Escherichia phage RB69	NP_861913.1	Hypothetical protein
48	dCMP deaminase	Escherichia phage vB_EcoM_EP32a	WPK28691.1	Nucleic acid metabolism
49	DAHP synthase I alpha	Enterobacter phage ENC9	UIW11250.1	Nucleic acid metabolism
50	hypothetical protein	Escherichia phage RB69	NP_861915.1	Hypothetical protein
51	hypothetical protein	Escherichia phage T4	YFO67222.1	Hypothetical protein
52	hypothetical protein	Escherichia phage AnYang	YP_010100354.1	Hypothetical protein
53	hypothetical protein	Escherichia phage XH12	XLY77938.1	Hypothetical protein
54	GIY-YIG catalytic domain protein	Escherichia phage XH12	XLY77939.1	Nucleic acid metabolism
55	polynucleotide kinase	Escherichia phage RB69	NP_861919.1	Nucleic acid metabolism
56	hypothetical protein	Raoultella phage vB_RorM_F4	YFW66104.1	Hypothetical protein
57	hypothetical protein	Escherichia phage RB69	NP_861921.1	Hypothetical protein
58	hypothetical protein	Escherichia phage KIT06	WCS66681.1	Hypothetical protein
59	hypothetical protein	Shigella phage JK36	QEG05808.1	Hypothetical protein
60	Rz-like spanin	Escherichia phage RB69	NP_861924.1	
61	inhibitor of host transcription	Escherichia phage JN02	QNI20693.1	Nucleic acid metabolism
62	RNA ligase and tail fiber protein attachment catalyst	Escherichia phage RB69	NP_861926.1	Nucleic acid metabolism
63	endonuclease	Escherichia phage RB69	NP_861927.1	Nucleic acid metabolism
64	ribonucleotide reductase of class Ia	Escherichia phage PNJ-6	WBM23469.1	Nucleic acid metabolism
65	NrdA-like aerobic NDP reductase large subunit	Escherichia phage RB69	NP_861929.1	Nucleic acid metabolism
66	HNH endonuclease	Escherichia phage vB_EcoM-RPN226	ULA51779.1	Nucleic acid metabolism
67	ribonucleoside-diphosphate reductase subunit alpha	Escherichia phage vB_EcoM-ZQ3	QWY13312.1	Nucleic acid metabolism
68	group I intron endonuclease	Escherichia phage vB_EcoM_WFL6982	QBQ76990.1	Nucleic acid metabolism
69	hypothetical protein	Escherichia phage RB69	NP_861930.1	Hypothetical protein
70	thymidylate synthase	Escherichia phage RB69	NP_861931.1	Nucleic acid metabolism
71	dihydrofolate reductase	Escherichia phage vB_EQ1	XPQ48375.1	Nucleic acid

ORF	Product	Source of Function	Accession	Functional category
				metabolism
72	hypothetical protein	Escherichia phage AV114	WPK34975.1	Hypothetical protein
73	hypothetical protein	Escherichia phage RB69	NP_861934.1	Hypothetical protein
74	single stranded DNA-binding protein	Escherichia phage vB_EcoP_EP32B	WPK28889.1	Nucleic acid metabolism
75	DNA helicase loader	Escherichia phage RB69	NP_861937.1	Nucleic acid metabolism
76	late promoter transcriptional regulator	Escherichia phage RB69	NP_861938.1	Nucleic acid metabolism
77	transcriptional regulator	Escherichia phage RB69	NP_861939.1	Nucleic acid metabolism
78	RnaseH	Escherichia phage RB69	NP_861940.1	Nucleic acid metabolism
79	long tail fiber proximal subunit	Escherichia phage vB_EcoM_NBG1	AWM11694.1	Structural protein
80	hypothetical protein	Escherichia coli O157 typing phage 13	AKE47003.1	Hypothetical protein
81	tail connector protein	Escherichia phage vB_EcoM_EP32a	WPK28660.1	Structural protein
82	tail fiber	Escherichia phage AV115	WPK35245.1	Structural protein
83	tail fiber assembly protein gp38 - phage TuIa	Enterobacteria phage TuIa	S13238	Structural protein
84	holin	Escherichia phage vB_EcoM_ESCO47	UPW39408.1	Lysis-associated protein
85	anti-sigma factor	Escherichia phage AV116	WPK35507.1	Nucleic acid metabolism
86	hypothetical protein	Escherichia phage Anabia	YDE38931.1	Hypothetical protein
87	hypothetical protein	Escherichia phage vB_EcoM-pJBK	UAW07030.1	Hypothetical protein
88	hypothetical protein	Escherichia phage AugustSocin	QXV76301.1	Hypothetical protein
89	hypothetical protein	Escherichia phage RB69	NP_861951.1	Hypothetical protein
90	hypothetical protein		YP_010093304.1	Hypothetical protein
91	hypothetical protein	vB_EcoM_VR25	YP_009210016.1	Hypothetical protein
92	MotA-like activator of middle period transcription	Escherichia phage RB69	NP_861954.1	Nucleic acid metabolism
93	DNA topoisomerase II	Escherichia phage RB69	NP_861955.1	Nucleic acid metabolism
94	hypothetical protein	Escherichia phage RB69	NP_861956.1	Hypothetical protein
95	Ac acridine resistance protein	Escherichia phage RB69	NP_861957.1	Host manipulation / anti-defense protein
96	Ndd-like nucleoid disruption protein	Escherichia phage RB69	NP_861958.1	Host manipulation / anti-defense protein
97	hypothetical protein	Escherichia phage RB69	NP_861959.1	Hypothetical protein
98	hypothetical protein	Escherichia phage RB69	NP_861960.1	Hypothetical protein
99	endonuclease	Escherichia phage vB_EcoP_EP32B	WPK29125.1	Nucleic acid metabolism
100	hypothetical protein	Escherichia phage RB69	NP_861962.1	Hypothetical protein
101	hypothetical protein	Escherichia phage PTK	QSL99358.1	Hypothetical protein
102	hypothetical protein	Escherichia coli O157 typing phage 13	AKE46981.1	Hypothetical protein
103	rIIA lysis inhibitor	Escherichia phage AV109	WPK33691.1	Lysis-associated protein
104	hypothetical protein	Shigella phage JK45	YP_010076081.1	Hypothetical

ORF	Product	Source of Function	Accession	Functional category
				protein
105	DNA topoisomerase II large subunit	Enterobacteria phage ATK47	YP_010088259.1	Nucleic acid metabolism
106	hypothetical protein	Escherichia phage RB69	NP_861695.1	Hypothetical protein
107	Zn-ribbon protein, partial	Escherichia phage vB_EcoM-pJBB	UYE95154.1	Nucleic acid metabolism
108	hypothetical protein	Escherichia phage RB69	NP_861697.1	Hypothetical protein
109	modifier of supressor tRNAs	Escherichia phage GADS24	WGG26123.1	Nucleic acid metabolism
110	transcriptional regulator	Escherichia phage vB_EcoM_PTE-Eco05	XUY70652.1	Nucleic acid metabolism
111	transcriptional regulator	Escherichia phage vB-Eco-KMB38	WQN06994.1	Nucleic acid metabolism
112	transcriptional regulator	Escherichia phage PNJ-9	XEN39152.1	Nucleic acid metabolism
113	exonuclease	Escherichia phage RB69	NP_861704.1	Nucleic acid metabolism
114	Dda-like helicase	Escherichia phage RB69	NP_861705.1	Nucleic acid metabolism
115	hypothetical protein	Escherichia phage RB69	NP_861706.1	Hypothetical protein
116	anti-termination	Escherichia phage AV115	WPK35282.1	Nucleic acid metabolism
117	ribosyltransferase	vB_EcoP_EP32B	WPK29104.1	Nucleic acid metabolism
118	hypothetical protein	Escherichia phage RB69	NP_861710.1	Hypothetical protein
119	hypothetical protein	Escherichia phage RB69	NP_861711.1	Hypothetical protein
120	hypothetical protein	Salmonella enterica subsp. enterica serovar Bareilly	EPJ2511899.1	Hypothetical protein
121	hypothetical protein	Escherichia phage PNJ-6	WBM23527.1	Hypothetical protein
122	hypothetical protein	Escherichia phage ST0	YP_009608380.1	Hypothetical protein
123	hypothetical protein	Escherichia phage RB69	NP_861716.1	Hypothetical protein
124	head decoration protein	Escherichia phage vB_EcoP_EP32B	WPK29096.1	Structural protein
125	dCTP pyrophosphatase	Escherichia phage vB_EcoP_EP32B	WPK29095.1	Nucleic acid metabolism
126	hypothetical protein	Escherichia phage RB69	NP_861719.1	Hypothetical protein
127	DNA primase	Escherichia phage RB69	NP_861720.1	Nucleic acid metabolism
128	hypothetical protein	Escherichia phage RB69	NP_861722.1	Hypothetical protein
129	hypothetical protein	Escherichia phage RB69	NP_861723.1	Hypothetical protein
130	hypothetical protein	Escherichia phage RB69	NP_861724.1	Hypothetical protein
131	spackle periplasmic protein	Escherichia phage SF-E2	YEN86918.1	Host manipulation / anti-defense protein
132	immunity to superinfection	Shigella phage CM8	YP_010075851.1	Host manipulation / anti-defense protein
133	hypothetical protein	Escherichia phage 55	WEW54109.1	Hypothetical protein
134	hypothetical protein	Escherichia phage RB69	NP_861728.1	Hypothetical protein
135	Dmd discriminator of mRNA degradation	Escherichia phage RB69	NP_861729.1	Host manipulation / anti-defense protein
136	hypothetical protein	Escherichia phage PCM001	WNN13560.1	Hypothetical protein

ORF	Product	Source of Function	Accession	Functional category
137	hypothetical protein	Escherichia phage RB69	NP_861731.1	Hypothetical protein
138	AAA family ATPase	Escherichia phage RB69	NP_861732.2	Nucleic acid metabolism
139	head formation protein	Escherichia phage vB_EcoM_SYGD1	QUD16034.1	Structural protein
140	DNA repair protein	Escherichia phage IME08	YP_003734188.1	Nucleic acid metabolism
141	thymidylate kinase	Escherichia phage RB69	NP_861735.1	Nucleic acid metabolism
142	hypothetical protein	Escherichia phage RB69	NP_861736.1	Hypothetical protein
143	thymidylate synthase	Enterobacter phage vB_EclM_CIP9	YP_009856035.1	Nucleic acid metabolism
144	hypothetical protein	Escherichia phage RB69	YFO67127.1	Hypothetical protein
145	peptidase	Escherichia phage OLB35	AYR03958.1	Nucleic acid metabolism
146	phosphoheptose isomerase	Escherichia phage RB69	NP_861741.1	Nucleic acid metabolism
147	hypothetical protein	Escherichia phage vB_EcoM_WFbE185	QBQ77554.1	Hypothetical protein
148	hypothetical protein	Escherichia phage CAU_ECP01	XLQ29113.1	Hypothetical protein
149	arabinose 5-phosphate isomerase	Escherichia phage RB69	NP_861745.1	Nucleic acid metabolism
150	DNA polymerase	Escherichia phage OLB35	AYR03965.1	Nucleic acid metabolism
151	translation repressor	Escherichia phage RB69	NP_861747.1	Host manipulation / anti-defense protein
152	clamp loader, small subunit	Shigella phage Sf25	AUV63460.1	Structural protein
153	AAA family ATPase	Escherichia coli	WP_015971446.1	Nucleic acid metabolism
154	DNA polymerase processivity factor	Shigella phage SHSML-52-1	YP_009288962.1	Nucleic acid metabolism
155	RNA polymerase binding	Escherichia phage RB69	NP_861751.1	Nucleic acid metabolism
156	hypothetical protein	Escherichia phage RB69	NP_861752.1	Hypothetical protein
157	homing endonuclease	Escherichia phage a20	WBF82730.1	Nucleic acid metabolism
158	SbcC-like subunit of palindrome specific endonuclease	Escherichia phage RB69	NP_861753.1	Nucleic acid metabolism
159	hypothetical protein	Escherichia phage vB_EcoP_EP32B	WPK29060.1	Hypothetical protein
160	hypothetical protein	Escherichia phage NHEP36	YFO67111.1	Hypothetical protein
161	recombination endonuclease subunit	Escherichia phage vB_EcoM-ZQ3	QWY13221.1	Nucleic acid metabolism
162	hypothetical protein	Escherichia phage RB69	NP_861757.1	Hypothetical protein
163	hypothetical protein	Escherichia phage RB69	NP_861758.1	Hypothetical protein
164	hypothetical protein	Escherichia phage vB_EcoM_ESCO47	UPW39331.1	Hypothetical protein
165	hypothetical protein	Escherichia phage RB69	NP_861760.1	Hypothetical protein
166	RNA polymerase sigma factor	Escherichia phage RB69	NP_861761.1	translational regulator
167	hypothetical protein	Escherichia phage RB69	NP_861762.1	Hypothetical protein
168	hypothetical protein	Escherichia phage RB69	NP_861763.1	Hypothetical protein
169	hypothetical protein	Escherichia phage vB_EcoM-pJBK	UAW07215.1	Hypothetical protein

ORF	Product	Source of Function	Accession	Functional category
170	hypothetical protein	Escherichia phage NHEP10	XUK82232.1	Hypothetical protein
171	hypothetical protein	Escherichia phage RB69	NP_861766.1	Hypothetical protein
172	hypothetical protein	Shigella phage Shf125875	YP_009100615.1	Hypothetical protein
173	hypothetical protein	Escherichia phage ChristianSchoenbein	QXV77015.1	Hypothetical protein
174	hypothetical protein	Escherichia phage vB_EcoP_EP32B	WPK29044.1	Hypothetical protein
175	hypothetical protein	Escherichia phage AV114	WPK35078.1	Hypothetical protein
176	hypothetical protein	Escherichia phage F2	YP_010068802.1	Hypothetical protein
177	putative glutaredoxin	vB_EcoM_PhAPEC2	YP_009056662.1	
178	hypothetical protein	Shigella phage CT01	UDY80467.1	Hypothetical protein
179	hypothetical protein	Shigella phage ESh25	URY13221.1	Hypothetical protein
180	hypothetical protein	vB_EcoM_PhAPEC2	YP_009056665.1	Hypothetical protein
181	anaerobic ribonucleotide reductase small subunit	Escherichia phage RB69	NP_861774.1	Nucleic acid metabolism
182	anaerobic ribonucleoside-triphosphate reductase, partial	Enterococcus faecalis	WP_411399594.1	Nucleic acid metabolism
183	intron-associated endonuclease 2	Escherichia phage 236Ecol005PP	WWT49648.1	Nucleic acid metabolism
184	NrdD-like anaerobic ribonucleotide reductase large subunit	Escherichia phage vB_EcoM_G8	YP_010069376.1	Nucleic acid metabolism
185	homing endonuclease	Escherichia phage Pu-Krd-SF3	XGU05876.1	Nucleic acid metabolism
186	endonuclease VII	Escherichia phage RB69	NP_861776.1	Nucleic acid metabolism
187	inhibitor of host Lon protease	Escherichia phage LF82-1	XUL00388.1	Host manipulation / anti-defense protein
188	hypothetical protein	Escherichia phage RB69	NP_861779.1	Hypothetical protein
189	hypothetical protein	Escherichia phage NRG-P0074	XKC24105.1	Hypothetical protein
190	hypothetical protein	Shgella phage phi25-307	YP_010093131.1	Hypothetical protein
191	hypothetical protein	Escherichia phage phiC120	YP_010090185.1	Hypothetical protein
192	phage-associated thioredoxin	Escherichia phage RB69	NP_861783.1	Nucleic acid metabolism
193	hypothetical protein	Escherichia phage RB69	NP_861784.1	Hypothetical protein
194	hypothetical protein	Escherichia phage RB69	NP_861785.1	Hypothetical protein
195	hypothetical protein	Escherichia phage HX01	YP_010246944.1	Hypothetical protein
196	thioredoxin	Escherichia phage vB_EcoP_EP32B	WPK29027.1	Nucleic acid metabolism
197	hypothetical protein	Escherichia phage RB69	NP_861788.1	Hypothetical protein
198	hypothetical protein	Escherichia phage RB69	NP_861789.1	Hypothetical protein
199	hypothetical protein	Escherichia phage vB_EcoM_SQ17	QVW29248.1	Hypothetical protein
200	hypothetical protein	Escherichia phage RB69	NP_861791.1	Hypothetical protein
201	hypothetical protein	Escherichia phage PECP20	WKM80990.1	Hypothetical protein

ORF	Product	Source of Function	Accession	Functional category
202	nucleotidyltransferase	Escherichia phage RB69	NP_861793.1	Nucleic acid metabolism
203	hypothetical protein	Escherichia phage JM10	XAO14206.1	Hypothetical protein
204	hypothetical protein	Escherichia phage RB69	NP_861795.1	Hypothetical protein
205	helicase loader	Escherichia phage RB69	NP_861796.1	Nucleic acid metabolism
206	hypothetical protein	Escherichia phage PNJ-6	WBM23607.1	Hypothetical protein
207	hypothetical protein	Escherichia phage RB69	NP_861797.1	Hypothetical protein
208	hypothetical protein	Escherichia phage RB69	NP_861798.1	Hypothetical protein
209	hypothetical protein	Escherichia phage vB_EcoM_WFbE185	QBQ77611.1	Hypothetical protein
210	hypothetical protein	Escherichia phage RB69	NP_861800.1	Hypothetical protein
211	thymidine kinase	Yersinia phage PYps14T	QLF85648.1	Nucleic acid metabolism
212	hypothetical protein	Klebsiella phage vB_KM5a1-KLB31	XOA00129.1	Hypothetical protein
213	hypothetical protein	Escherichia phage RB69	NP_861802.1	Hypothetical protein
214	hypothetical protein	Escherichia phage a20	WBF82782.1	Hypothetical protein
215	hypothetical protein	Escherichia phage W143	QWV60457.1	Hypothetical protein
216	macro domain-containing protein	Salmonella enterica subsp. enterica serovar Bareilly	EPJ2511789.1	Nucleic acid metabolism
217	valyl tRNA synthetase modifier	Escherichia phage RB69	NP_861804.1	Host manipulation / anti-defense protein
218	hypothetical protein	Escherichia phage ST0]	YP_009608290.1	Hypothetical protein
219	endoribonuclease	Escherichia phage RB69	NP_861806.1	Nucleic acid metabolism
220	hypothetical protein	Escherichia phage vB_EcoP_EP32B	WPK29006.1	Hypothetical protein
221	hypothetical protein	Escherichia phage RB69	NP_861808.1	Hypothetical protein
222	hypothetical protein	Escherichia phage RB69	NP_861809.1	Hypothetical protein
223	autonomous glycyl radical cofactor GrcA	Escherichia phage T4	NP_049730.1	Nucleic acid metabolism
224	hypothetical protein	Escherichia phage RB69	NP_861811.1	Hypothetical protein
225	hypothetical protein	Escherichia phage vB_EcoP_EP32B	WPK29002.1	Hypothetical protein
226	hypothetical protein	Shigella phage JK42	QEG06238.1	Hypothetical protein
227	putative pyrimidine dimer DNA glycosylase	Escherichia phage vB_EcoM_G53	QBO65262.1	Nucleic acid metabolism
228	internal head protein	Escherichia phage HX01	YP_010246909.1	Structural protein
229	glycoside hydrolase protein	Escherichia phage RB69	NP_861818.1	Lysis-associated protein
230	nudix hydrolase	Escherichia phage RB69	NP_861819.1	Nucleic acid metabolism
231	hypothetical protein	Escherichia phage AV109	WPK33811.1	Hypothetical protein
232	hypothetical protein	Escherichia phage a20	WBF82804.1	Hypothetical protein
233	hypothetical protein	Escherichia phage RB69	NP_861823.1	Hypothetical protein
234	hypothetical protein	Escherichia phage RB69	NP_861824.1	Hypothetical protein

ORF	Product	Source of Function	Accession	Functional category
235	hypothetical protein	Escherichia phage RB69	NP_861825.1	Hypothetical protein
236	hypothetical protein	Shigella phage phi25-307	YP_010093179.1	Hypothetical protein
237	hypothetical protein	Escherichia phage RB69	NP_861827.1	Hypothetical protein
238	hypothetical protein	Escherichia phage RB69	NP_861829.1	Hypothetical protein
239	hypothetical protein	Escherichia phage RB69	NP_861830.1	Hypothetical protein
240	hypothetical protein	Escherichia phage AV110	WPK34080.1	Hypothetical protein
241	hypothetical protein	Escherichia phage vB_EcoM-ZQ3	QWY13412.1	Hypothetical protein
242	hypothetical protein	Escherichia phage RB69	NP_861833.1	Hypothetical protein
243	hypothetical protein	Escherichia phage RB69	NP_861834.1	Hypothetical protein
244	hypothetical protein	Escherichia phage RB69	NP_861836.1	Hypothetical protein
245	hypothetical protein	Escherichia phage QL01	YP_009202866.1	Hypothetical protein
246	hypothetical protein	Escherichia phage vB_EcoM_EP32a	WPK28766.1	Hypothetical protein
247	hypothetical protein	Escherichia phage vB_EcoM_WFL6982	QBQ76898.1	Hypothetical protein
248	hypothetical protein	Escherichia phage PHB12	QBP05759.1	Hypothetical protein
249	hypothetical protein	Escherichia phage vB_EcoM_EP32a	WPK28764.1	Hypothetical protein
250	hypothetical protein	Escherichia phage vB_EcoM_HZ_ZJUN4	WVX92669.1	Hypothetical protein
251	hypothetical protein	Escherichia phage vB_EcoP_EP32B	WPK28986.1	Hypothetical protein
252	hypothetical protein	Shigella phage JK45	YP_010076228.1	Hypothetical protein
253	hypothetical protein	Escherichia phage AV111	WPK34362.1	Hypothetical protein
254	chaperone for tail fiber formation	Escherichia phage vB_EcoM_ESCO47	UPW39254.1	Assembly accessory protein
255	deoxynucleoside monophosphate kinase	Escherichia phage RB69	NP_861849.1	Nucleic acid metabolism
256	tail completion and sheath stabilizer protein	Escherichia phage RB69	NP_861850.1	Structural protein
257	DNA end protector protein	Escherichia phage vB_EcoP_EP32B	WPK28981.1	Nucleic acid metabolism
258	homing endonuclease	Yersinia phage vB_YenM_P8	XAO54378.1	Nucleic acid metabolism
259	head closure	Escherichia phage RB69	NP_861852.1	Structural protein
260	baseplate wedge subunit	Escherichia phage F2	YP_010068994.1	Structural protein
261	tail lysozyme	Escherichia phage RB69	NP_861854.1	Lysis-associated protein
262	hypothetical protein	Escherichia phage F2	YP_010068992.1	Hypothetical protein
263	hypothetical protein	Escherichia phage RB69	NP_861855.1	Hypothetical protein
264	PAAR motif of membran proteins	Escherichia phage AV110	WPK34103.1	Host manipulation / anti-defense protein
265	baseplate wedge subunit	Escherichia phage RB69	NP_861857.1	Structural protein
266	baseplate wedge subunit	Escherichia phage vB_EcoM_ESCO47	UPW39244.1	Structural protein
267	baseplate wedge subunit	Escherichia phage vB_Eco_F31	WBF80724.1	Structural protein
268	putative baseplate wedge tail fiber connector	Escherichia phage vB_EcoM_ESCO47	UPW39242.1	Structural protein

ORF	Product	Source of Function	Accession	Functional category
269	baseplate wedge subunit and tail pin	Escherichia phage vB_EcoM_ESCO47	UPW39241.1	Structural protein
270	baseplate wedge subunit and tail pin	Escherichia phage vB_EcoM_ESCO47	UPW39240.1	Structural protein
271	tail fiber protein	Escherichia coli	WP_236304025.1	Structural protein
272	fibritin neck whiskers protein	Escherichia phage vB_EcoM_ESCO47	UPW39486.1	Structural protein
273	head-tail adaptor Ad2	Escherichia phage RB69	NP_861865.1	Structural protein
274	putative endonuclease	Escherichia phage moskry	QHR76628.1	Nucleic acid metabolism
275	head closure Hc2	Escherichia phage ST0	YP_009608501.1	Structural protein
276	tail sheath stabilizer	Escherichia phage RB69	NP_861867.1	Structural protein

Functional annotation and characteristics of coding sequences in phage vB_EcoM_S37.

Table S3

Functional annotation and characteristics of coding sequences in phage vB_EcoM_S37.

ORF	Product	Source of Function	Accession	Functional category
1	ocr-like anti-restriction	Escherichia phage T7	NP_041954.1	Effector protein
2	hypothetical protein	Acidobacteriota bacterium	MCH7824682.1	Hypothetical protein
3	serine/threonine kinase	Yersinia phage	UYE95485.1	Effector protein
4	T7 RNA polymerase	Expression vector pSEVA 221TadAT7RNAP	QGQ59661.1	DNA replication and trans cription
5	hypothetical protein	Escherichia phage T7	NP_041961.1	Hypothetical protein
6	phage T7 gene 1.2 fa mily protein	Edwardsiella phage PVN0 9	QXV72976.1	Effector protein
7	DNA ligase	Escherichia phage CICC 80001	YP_009152468.1	DNA replication and trans cription
8	hypothetical protein	Escherichia phage HZP2	YP_009820206.1	Hypothetical protein
9	AAA-ATP protein	Serratia phage Pila	QFG06785.1	DNA replication and trans cription
10	RNA polymerase inhi bitor	Escherichia phage T7	2LMC_A	Effector protein
11	Gp2.5-like ssDNA bin ding protein and ssD NA annealing protein	Escherichia phage T7	NP_041970.1	Effector protein
12	endonuclease	Yersinia phage phiA1122	NP_848275.1	DNA replication and trans cription
13	lysozyme	Escherichia phage PGX1	WGL32416.1	Host Lysis
14	DNA primase/helicase	Escherichia phage T7	1E0J_A	DNA replication and trans cription
15	hypothetical protein	Escherichia phage T7	NP_041979.1	Hypothetical protein
16	hypothetical protein	Escherichia phage T7	NP_041980.1	Hypothetical protein
17	hypothetical protein	Escherichia phage T7	UJQ71070.1	Hypothetical protein
18	DNA polymerase I	Yersinia phage phiA1122	NP_848283.1	DNA replication and trans cription
19	DUF2675 domain - c ontaining protein	Escherichia phage T7	NP_041985.1	Effector protein
20	Gp5.9-like inihibitor o f recBCD nuclease	Escherichia phage HZP2	YP_009820221.1	Effector protein
21	exonuclease	Escherichia phage T7	NP_041988.1	DNA replication and trans cription
22	DUF2717 domain-cont aining protein	Yersinia phage phiA1122	NP_848289.1	Unknown protein
23	DUF5476 family prote in	Escherichia phage T7	NP_041991.1	Unknown protein
24	host range and adsorp	Yersinia phage phiA1122	NP_848291.1	Effector protein

ORF	Product	Source of Function	Accession	Functional category
	tion protein			
25	host range and adsorption protein	Enterobacter phage EcpYZU01	YDB53592.1	Hypothetical protein
26	Chain A of Portal Protein	Escherichia phage T7	6QX5_A	Structural protein
27	Head assembly	Yersinia phage phiA1122	NP_848295.1	Structural protein
28	major head protein	Yersinia phage phiA1122	NP_848296.1	Structural protein
29	head protein	Yersinia phage fPS	YP_009799209.1	Structural protein
30	Tail tubular protein gp11	Escherichia phage T7	6R21_M	Structural protein
31	tail protein	Escherichia phage T7	NP_042000.1	Structural protein
32	internal virion protein	Escherichia phage T7	NP_042001.1	Structural protein
33	internal virion protein	Escherichia phage T7	8E4G_a	Structural protein
34	internal virion protein	Escherichia phage HZP2	YP_009820235.1	Structural protein
35	internal virion protein	Cedecea phage Yanou	UTQ78081.1	Structural protein
36	Tail fiber protein	Salmonella phage Sal_R1S	WZP35960.1	Structural protein
37	hypothetical protein	Citrobacter phage vB_CroP_CrRp3	YP_009797799.1	Hypothetical protein
38	phage holin family protein	Pseudomonas aeruginosa	WP_434006990.1	Host Lysis
39	terminase small subunit	Escherichia phage T7	AAC31982.1	DNA packaging protein
40	Rz-like spanin	Escherichia phageHZP2	YP_009820196.1	Host Lysis
41	Terminase large subunit	Escherichia phage T7	4BIJ_A	DNA packaging protein
